# Synthesis of Pseudooligosaccharides Related to the Capsular Phosphoglycan of *Haemophilus influenzae* Type *a*

**DOI:** 10.3390/molecules28155688

**Published:** 2023-07-27

**Authors:** Anastasia A. Kamneva, Dmitry V. Yashunsky, Elena A. Khatuntseva, Nikolay E. Nifantiev

**Affiliations:** N. D. Zelinsky Institute of Organic Chemistry, Leninsky pr. 47, Moscow 119991, Russia; nastyakamneva@icloud.com (A.A.K.); yashunsky1959@yandex.ru (D.V.Y.); glyco@ioc.ac.ru (E.A.K.)

**Keywords:** *Haemophilus influenzae* type *a*, phosphoglycan, pseudooligosaccharide, biotinylated neoglycoconjugate, H-phosphonate

## Abstract

Synthesis of spacer-armed pseudodi-, pseudotetra-, and pseudohexasaccharides related to the capsular phosphoglycan of *Haemophilus influenzae* type *a*, the second most virulent serotype of *H. influenzae* (after type *b*), was performed for the first time via iterative chain elongation using H-phosphonate chemistry for the formation of inter-unit phosphodiester bridges. These compounds were prepared for the design of neoglycoconjugates, as exemplified by the transformation of the obtained pseudohexasaccharide derivative into a biotinylated glycoconjugate suitable for use in immunological studies, particularly in diagnostic screening systems as a coating antigen for streptavidin-coated plates and chip slides.

## 1. Introduction

Among the numerous types of the extracellular pathogenic bacteria *Haemophilus influenzae*, the six encapsulated types are referred to as *H. influenzae* serotypes *a*–*f* [1]. Each of these bacteria has a unique glycan capsule which is associated with virulence and invasive bacterial infections [2]. In the pre-vaccine era, *H. influenzae* type *b* (*Hib*) contributed substantially to the burden of meningitis and other life-threatening infections in young children in the USA and Europe [3,4], while the other serotypes were considered non-invasive. After the conjugate *Hib* vaccine [5,6,7,8,9,10] was introduced into childhood immunization schedules, an epidemiological shift to other strains belonging to serotypes *H. influenzae* type *a* (*Hia*) [11,12,13,14,15,16], *e* [11,17], and *f* [11,18] was observed worldwide.

*Hia* was found to be the second most virulent serotype of *H. influenzae* (after *Hib*) [19]. It is a causative agent of meningitis, bacteremia, and pneumonia and affects mostly children under two years of age [15]. The rate of serotype replacement with *Hia* is impressive: in Brazil, a year after the implementation of *Hib* immunization, the surveillance for *H. influenzae* meningitis cases showed an eight-fold increase in the incidence of *Hia* meningitis [16]. Therefore, there is a clear need for a *Hia* testing system to monitor capsule replacement of *Hia* in countries where the *Hib* vaccine is a part of the routine childhood immunization schedule. As capsular glycans (CGs) are the principal bacterial antigens that induce high levels of serum immunoglobulins [20], this testing system is expected to be a convenient serological surveillance tool for the identification and monitoring of the disease agent.

**Figure 1:**
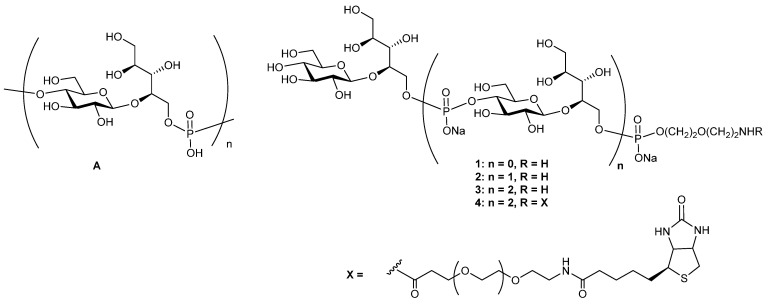
The structure of Hia CG (A), synthesized spacer-armed oligosaccharides **1**–**3**, and biotinylated glycoconjugate **4**.

The structure of *Hia* CG was published in 1977 [21] and consists of a linear phosphoglycan (Figure 1, structure A) with a β-d-Glcp-(1→4)-d-Rib-ol-5-PO_4_→4- repeating unit. In 1988, two isomeric pseudodisaccharides related to a *Hia* CG repeating unit [22] phosphorylated either at O-3 of the glucose residue or O-5 of the ribitol residue with triazol-activated phosphate precursors [23] were prepared. Also, the synthesis of a series of *Hia* CG-related pseudooligosaccharides through the use of the phosphoramidite approach for the formation of inter-unit phosphodiester bridges was described in a PhD thesis in 2020 [24]. However, a convenient synthetic approach to *Hia* CG-related pseudooligosaccharides has not been yet reported.

A characteristic feature of the structure of *Hia* CG is the presence of a phosphodiester bridge between the glucose and the ribitol unit. Currently, there are two main approaches for the synthesis of carbohydrate derivatives with phosphodiester fragments: the H-phosphonate [25] and the phosphoramidite [26] methods. The latter was used in the published synthesis of a pseudodisaccharide related to a *Hia* CG repeating unit [22].

In the present paper, we report the synthesis of a series of spacer-armed pseudodi-, pseudotetra-, and pseudohexasaccharide derivatives (**1**–**3**), which are structurally related to one, two, and three repeating units of the *Hia* CG, based on a block-wise chain assembling scheme and the use of H-phosphonate chemistry [27] for the formation of inter-unit phosphodiester bridges. As compared to the phosphoramidite approach, the H-phosphonate method [27] has the following principal advantages: (a) relative simplicity and availability of reagents (phosphorous acid, pivaloyl chloride, iodine), (b) the formation of only one diasteriomeric product and not a mixture of diastereomers, and (c) no need for an additional P-deprotection step. Therefore, the H-phosphonate approach seems to be more preferable for the synthesis of molecules with more than one phosphodiester fragment. Using H-phosphonate chemistry, compounds **1**–**3** and a biotinylated pseudohexasaccharide derivative **4** (Figure 1) were prepared with a view to develop an affordable and effective screening tool for fast and unambiguous detection of *Hia*-associated invasive diseases.

## 2. Results and Discussion

**Scheme 1:**
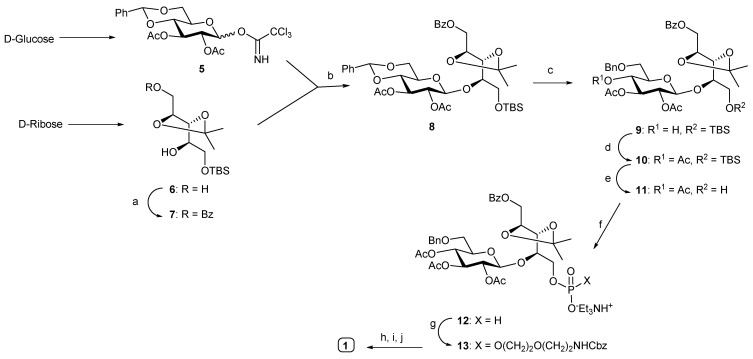
Synthesis of pseudodisaccharide **1**. Reagents and conditions: (a) BzCl, Py, 77%; (b) TMSOTf, CH_2_Cl_2_, 60%; (c) Me_3_N·BH_3_, AlCl_3_, H_2_O, THF, 64%; (d) Ac_2_O, DMAP, Py; (e) HF_aq_, Py, 60 °C, 97% over two steps; (f) H_3_PO_3_, PivCl, Py, 74%; (g) CbzNH(CH_2_)_2_O(CH_2_)_2_OH, PivCl, Py, then I_2_, H_2_O, 34%; (h) TFA_aq._ ultrasonication; (i) 0.09M NaOMe/MeOH; (j) H_2_ gas, Pd(OH)_2_/C, AcOH, MeOH, 36% over three steps.

Previously described imidate **5** [28] and ribitol derivative **6** [29,30] (Scheme 1) were used as starting materials for the preparation of target compounds **1**–**3**. Regioselective benzoylation of diol **6** transformed it into its 1-*O*-benzoylated derivative **7**, which was then glycosylated by imidate **5** [28] in the presence of TMSOTf to obtain pseudodisaccharide product **8**. β-Configuration of the newly formed glycosidic bond in compound **8** was confirmed by the characteristic value of the coupling constant (*J*_1,2_ 7.8 Hz). Regioselective reductive opening of a 4,6-benzylidene acetal in glycoside **8** (δ PhCH 5.49 ppm) under the action of Me_3_N∙BH_3_–AlCl_3_–H_2_O–THF [31] readily afforded the key building block **9** (δ _C-4_ 71.4, δ _C-6_ 70.6 ppm) at a yield of 64%. The presence of the benzyl group at O-6 and the location of the free OH- group at C-4 of the glucose unit was unambiguously confirmed by disappearance of the signals of the 4,6-*O*-benzylidene group, the downfield displacement of the chemical shift of C-6 by 3 ppm, and the upfield displacement of the chemical shift of C-4 by 7 ppm in the ^13^C NMR spectrum (see Section 3). O-Acetylation of **9** and desilylation of the thus obtained acetate **10** provided alcohol **11** at an excellent yield (97% over 2 steps).

The H-phosphonate building block **12** was prepared by the treatment of primary alcohol **11** with a pyrophosphonate obtained in situ from 2 moles of phosphonic acid and 1 mole of pivaloyl chloride to avoid the formation of a diester by-product [32]. Condensation of H-phosphonate **12** with CbzNH(CH_2_)_2_O(CH_2_)_2_OH in Py in the presence of Et_3_N as an organic base and PivCl as a condensing agent, followed by oxidation of the intermediate product with I_2_ [27], provided the corresponding target pseudodisaccharide **13** at 74% yield. Removal of its isopropylidene group by hydrolysis in aq. trifluoroacetic acid followed by alkaline deacetylation and hydrogenolysis of the thus formed phosphodiester **13** afforded the spacer-armed monomer **1** at an overall 36% yield (Scheme 1).

**Scheme 2:**
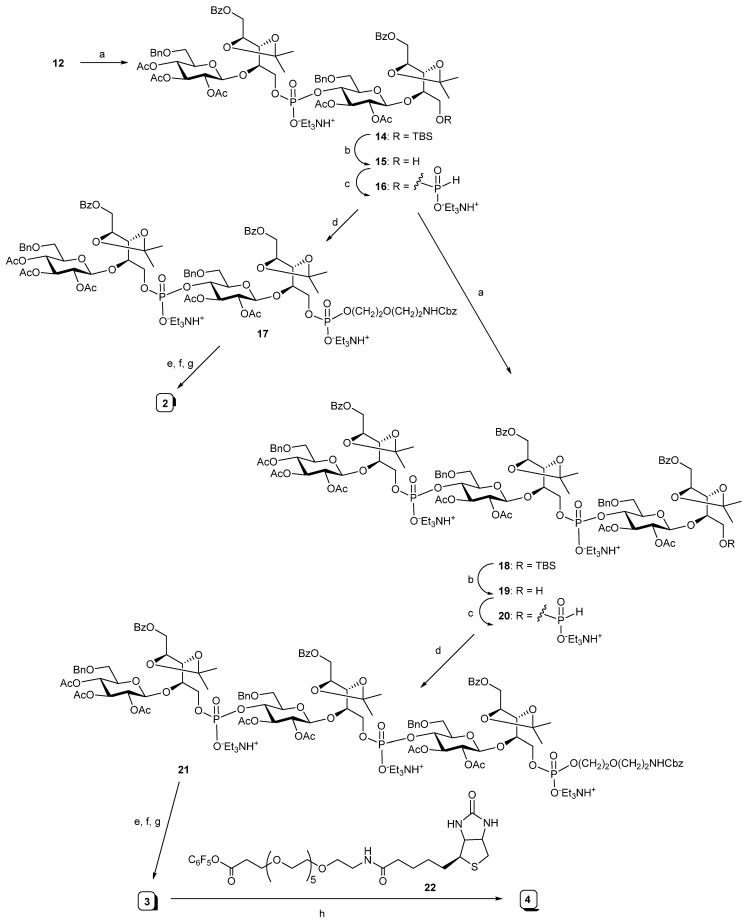
Synthesis of pseudotetrasaccharide **2**, pseudohexasaccharide **3,** and biotinylated derivative **4**. Reagents and conditions: (a) **9**, PivCl, Py, then I_2_, H_2_O, yield of 77% for **14** and 45% for **18**; (b) HF_aq_, Py, 60 °C, yield of 90% for **15** and 90% for **18**; (c) H_3_PO_3_, PivCl, Py, yield of 96% for **16**—and 71% for **20**; (d) CbzNH(CH_2_)_2_O(CH_2_)_2_OH, PivCl, Py, then I_2_, H_2_O, yield of 54% for **17**—and 70% for **21**; (e) TFA_aq._ ultrasonication; (f) 0.09M NaOMe/MeOH; (g) H_2_ gas, Pd(OH)_2_/C, AcOH, MeOH, yield of 59% for **2** and 90% for **3** over three steps; (h) Et_3_N, DMF, 70%.

In order to assemble compound **2**, phosphonylation of the alcohol **9** with H-phosphonate **12** in the presence of PivCl/Py and subsequent oxidation were performed to obtain phosphodiester **14** at a 77% yield, which was readily desilylated to produce the primary alcohol **15** (Scheme 2). Transformation of alcohol **15** into H-phosphonate **16** and the following condensation with CbzNH(CH_2_)_2_O(CH_2_)_2_OH resulted in the formation of the spacer-armed pseudotetrasaccharide **17**. Removal of the protective groups, as described above for the preparation of the pseudidisaccharide **1**, provided the target pseudotetrasaccharide **2** at a 59% yield.

The preparation of compound **3** was performed using the [4+2]-coupling strategy. Condensation of pseudo-tetrasaccharide H-phosphonate **16** and alcohol **9** followed by the oxidation step provided pseudohexasaccharide **18** at a moderate yield of 45% (Scheme 2). The subsequent desilylation step afforded alcohol **19**, which was transformed into H-phosphonate **20**. Its condensation with CbzNH(CH_2_)_2_O(CH_2_)_2_OH in the presence of PivCl/Py and the following oxidation afforded the spacer-armed pseudohexasaccharide **21** (44% over three steps). Total deprotection provided the target trimer **3** at 90% yield.

Treatment of pseudohexasaccharide **3** with biotin-containing pentafluorophenyl ester **22** [33] resulted in the formation of the target neoglycoconjugate **4** at a yield of 70%. The structure of the thus synthesized compounds **1**–**4** was confirmed by the combination of HRMS and NMR spectroscopy. In particular, the ^1^H, ^13^C, and ^31^P NMR spectra of these products contained full series of signals related to the spacer group, β-d-glucose, and ribitol units connected via (1→2)-bonds and the phosphodiester bridge (see Section 3.2.7, Section 3.2.12, Section 3.2.15 and Section 3.2.16). β-Configuration of the glycosidic bond in Glc (1→4)Rib was confirmed by characteristic J_1,2_ coupling constant values between 7.8 and 8.7 Hz in the ^1^H NMR spectra for all protected and deprotected derivatives. Also, for mono-, di-, and tri-phosphodiester derivatives, the corresponding signals in ^31^P NMR spectra were observed in the expected area of ~0 ppm.

## 3. Materials and Methods

### 3.1. General Information

Chemicals were purchased from Acros, Fluka, or Aldrich and used without further purification. All solvents were purified according to standard protocols. All reactions involving air- or moisture-sensitive reagents were carried out using dry solvents under an Ar atmosphere. TLC was performed on Silica Gel 60 F254 plates (E. Merck), and visualization was either accomplished using UV light or via charring at ~150 °C with a 20:1 mixture of 15% (*v*/*v*) H_3_PO_4_ in water and 4% orcinol in ethanol. Silica gel column chromatography was performed with Silica Gel 60 (40–63 μm, E. Merck). Gel filtration was performed on a TSK HW-40 column (400 × 17 mm) through elution with 0.1N CH_3_COOH at a flow rate of 0.6 mL/min. NMR spectra were recorded on Bruker AM 300 (300 MHz) and Bruker Avance 600 spectrometers. Assignment in ^1^H and ^13^C NMR spectra was performed using different 2D experiments (e.g., COSY, NOESY, HSQC). Chemical shifts are presented in ppm with reference to the solvent residual peaks used as a standard (δ 7.27 for chloroform in ^1^H NMR and δ 77.0 for ^13^C NMR). HRMS (ESI) spectra were obtained on a MicrOTOF II (Bruker Daltonics) instrument in positive or negative modes. Optical rotations were measured using a JASCO DIP-360 polarimeter at 16 °C in CHCl_3_.

### 3.2. Synthesis of Compounds ***7***, ***8***, ***10***, ***11***, ***12***, ***13***, ***1***, ***14***, ***15***, ***16***, ***17***, ***2***, ***18***, ***21***, ***3***, and ***4***

#### 3.2.1. 1-*O*-Benzoyl-5-*O*-(tert-butyldimethylsilyl)-2,3-*O*-isopropylidene-d-ribitol (**7**)

To a solution of **6** (3.4 g, 15 mmol) in Py (20 mL), BzCl (1.9 mL, 16.5 mmol) was added. The mixture was stirred for 2 h at rt, and the reaction mixture was then diluted with CH_2_Cl_2_ and washed with 1M HCl, water, and satd NaHCO_3_. The organic layer was concentrated in vacuo and the column chromatography provided **7** (3.5 g, 77%) as a colorless oil (see Supplementary Materials, p. 3). ${\left[\alpha \right]}_{D}^{16}$ −18.0 (*c* 1, CHCl_3_). R_f_ = 0.8 (Tol-EtOAc 2:1). ^1^H NMR (300 MHz, CDCl_3_) δ −0.1 (s, 6H, -Si(C*H*_3_)_2_), 0.75 (s, 9H, -C(C*H*_3_)_3_), 1.25 and 1.35 (both s, 6H, *Me*_2_C), 2.55 (br s, 1H, O*H*), 3.61 (dd, 1H, *J*_4,5_ = 4.3 Hz, *J*_5,6_ = 5.2 Hz, H-5b), 3.69 (t, 1H, *J*_3,4_ = *J*_4,5_ = 7.9 Hz, H-4), 3.77 (dd, 1H, *J*_4,5_ = 2.9 Hz, *J*_5,6_ = 9.6 Hz, H-5a), 4.03 (dd, 1H, *J*_2,3_ = 6.1 Hz, *J*_3,4_ = 9.3 Hz, H-3), 4.37 (dd, 1H, *J*_1,2_ = 7.2 Hz, *J*_gem_ = 11.5 Hz, H-1b), 4.51–4.43 (m, 1H, H-2), 4.67 (dd, 1H, *J*_1,2_ = 3.0 Hz, *J*_gem_ = 11.5 Hz, H-1a), 8.02–7.29 (m, 5H, Ph). ^13^C NMR (75 MHz, CDCl_3_) δ −5.3 and −5.4 (-Si(*C*H_3_)_2_), 25.5 and 27.9 (*Me*_2_C), 25.9 (-*C*(CH_3_)_3_), 64.0 (C-1), 64.5 (C-5), 69.3 (C-4), 75.7 (C-2), 76.0 (C-3), 109.2 (Me_2_*C*), 128.3, 129.8, and 132.9 (Ph), 166.5 (Ph*C*(O)O-). HRMS (ESI): calcd *m*/*z* for [M + Na]^+^ C_21_H_34_O_6_S 433.2017, found 433.2011.

#### 3.2.2. 1-*O*-Benzoyl-4-*O*-(4,6-*O*-benzylidene-2,3-di-*O*-acetyl-β-d-glucopyranosyl)-5-*O*-(tert-butyldimethylsilyl)-2,3-*O*-isopropylidene-d-ribitol (**8**)

To a mixture of compound **5** (100 mg, 0.2 mmol) and acceptor 7 (70 mg, 0.17 mmol) in anhydrous CH_2_Cl_2_ (1 mL), TMSOTf (1.1 μL, 6 μmol, 3% mol) was added. The mixture was stirred for 20 min at rt until full conversion of the starting material (TLC control), and the mixture was then neutralized with Et_3_N and concentrated in vacuo. The residue was purified by column chromatography to obtain product **8** (76 mg, 60%) as a white amorphous powder (see Supplementary Materials, p. 5). ${\left[\alpha \right]}_{D}^{16}$ −0.9 (*c* 1, CHCl_3_). *R*_f_ = 0.48 (toluene:EtOAc 5:1). ^1^H NMR (300 MHz, CDCl_3_) δ 0.08 and 0.09 (both s, 6H, -Si(C*H*_3_)_2_), 0.88 (s, 9H, -C(C*H*_3_)_3_), 1.36 and 1.47 (both s, 6H, *Me*_2_C), 2.06 and 2.08 (both s, 6H, -C(O)OC*H*_3_), 3.35–3.45 (m, 1H, H-5’), 3.64 (t, 1H, *J_3,4_ = J*_4,5_ = 9.6 Hz, H-4′), 3.69–3.83 (m, 2H, H-5b, H-6′b), 4.02–4.10 (m, 1H, H-6′a), 4.10–4.25 (m, 3H, H-5a, H-2, H-3), 4.46–4.53 (m, 1H, H-4), 4.57 (d, 1H, *J*_1,2_ = 5.9 Hz, *J_gem_* = 11.4 Hz, H-1b), 4.65 (dd, 1H, *J*_1,2_ = 2.3 Hz, *J_gem_* = 11.4 Hz, H-1a), 4.92–5.01 (m, 1H, H-2′), 5.17 (d, 1H, *J*_1,2_ = 7.8 Hz, H-1′), 5.28 (t, 1H, *J*_2,3_ = *J*_3,4_ = 9.5 Hz, H-3′), 5.49 (s, 1H, PhC*H*), 7.25–8.20 (m, 10H, Ph). ^13^C NMR (75 MHz, CDCl_3_) δ −5.5 and −5.4 (-Si(*C*H_3_)_2_), 18.2 and 20.8 (-C(O)O*C*H_3_), 25.5 and 27.6 (*Me*_2_C), 25.8 (-C(*C*H_3_)_3_), 63.9 (C-5), 64.4 (C-1), 66.3 (C-5’), 68.4 (C-6′), 71.9 (C-3′), 72.5 (C-2′), 74.2 and 74.8 (C-2, C-3), 75.4 (C-4), 78.4 (C-4′), 98.3 (C-1′), 108.9 (Me_2_*C*), 126.1, 126.2, 128.2, 128.4, 129.1, 129.6, 129.8, 130.3, 132.9, and 136.9 (Ph), 166.4 (PhC(O)O-), 169.7 and 170.2 (-O*C*(O)CH_3_). HRMS (ESI): calcd *m*/*z* for [M + Na]^+^ C_38_H_52_O_13_Si 767.3069, found 767.3070.

#### 3.2.3. 1-*O*-Benzoyl-4-*O*-(6-*O*-benzyl-2,3-di-*O*-acetyl-β-d-glucopyranosyl)-5-*O*-(tert-butyldimethylsilyl)-2,3-*O*-isopropylidene-d-ribitol (**10**)

A solution of **8** (1.4 g, 1.9 mmol) in THF (28 mL) was cooled to 0 °C, and Me_3_N·BH_3_ (562 mg, 7.7 mmol), AlCl_3_ (1.52 g, 11.4 mmol), and H_2_O (68 μL, 3.8 mmol) were then added. The reaction mixture was stirred at rt for 3 h, diluted with CH_2_Cl_2_, and washed with 1 M HCl, water, and satd NaHCO_3_. The organic layer was concentrated in vacuo and flash column chromatography of the residue provided product **9** (980 mg, 64%) as a colorless oil (see Supplementary Materials, p. 7). ${\left[\alpha \right]}_{D}^{16}$ −15.1 (*c* 1, CHCl_3_). R_f_ = 0.22 (toluene:EtOAc 4:1). ^1^H NMR (300 MHz, CDCl_3_) δ 0.10 and 0.11 (both s, 6H, -Si(C*H*_3_)_2_), 0.94 (s, 9H, -C(C*H*_3_)_3_), 1.36 and 1.46 (both s, 6H, *Me*_2_C), 2.06 and 2.08 (both s, 6H, -C(O)OC*H*_3_), 3.13 (br s, 1H, O*H*), 3.45–3.51 (m, 1H, H-5’), 3.69–3.81 (m, 4H, H-4′, H-5b, H-6′ab), 4.04 (dd, 1H, *J*_4,5_ = 11.5 Hz, *J*_5,6_ = 1.4 Hz, H-5a), 4.07–4.20 (m, 2H, H-2, H-3), 4.47–4.64 (m, 4H, H-1b, H-4, -C*H*_2_Ph), 4.69 (dd, 1H, *J*_1,2_ = 11.5 Hz, *J_gem_* = 2.3 Hz, H-1a), 4.90 (dd, 1H, *J*_1,2_ = 7.8 Hz, *J*_2,3_ = 9.8 Hz, H-2′), 5.02–5.08 (m, 2H, H-1′, H-3′). ^13^C NMR (75 MHz, CDCl_3_) δ −5.5 and −5.4 (-Si(*C*H_3_)_2_), 20.8 and 20.9 (-C(O)O*CH_3_*), 25.6 and 27.7 (*Me*_2_C), 25.9 (-C(*C*H_3_)_3_), 63.8 (C-5), 64.3 (C-1), 70.6 (C-6′), 71.4 (C-4′), 73.5 (5‘), 73.8 (-*C*H_2_Ph), 74.3 and 74.7 (C-2, C-3), 75.4 and 75.5 (C-3′, C-4), 97.9 (C-1′), 108.9 (Me_2_*C*), 127.8, 127.9, 128.3, 128.4, 129.8, 130.3, and 132.8 (Ph), 166.4 (Ph*C* (O)O-), 169.7 and 171.1 (-*C*(O)OCH_3_). HRMS (ESI): calcd *m*/*z* for [M + Na]^+^ C_38_H_54_O_13_Si 769.3226, found 769.3225.

#### 3.2.4. 1-*O*-Benzoyl-4-*O*-(6-*O*-benzyl-2,3,4-tri-*O*-acetyl-β-d-glucopyrano-syl)-2,3-*O*-isopropylidene-d-ribitol (**11**)

To a solution of **9** (500 mg, 0.67 mmol) in a mixture of Py:Ac_2_O 2:1 (1.5 mL), DMAP (10 mg, 82 μmol) was added. The reaction mixture was stirred at rt for 1 h, and it was then quenched with MeOH, diluted with CH_2_Cl_2_, and washed with 1M HCl, water, and brine. The organic layer was concentrated in vacuo to provide the crude **10**. The residue was dissolved in a mixture of Py:HF (90% aq.) at a ratio of 6:1 (5.8 mL) in a plastic centrifuge tube. The reaction mixture was heated to 60 °C and stirred for 2 h until the starting material disappeared (TLC control). It was then diluted with CH_2_Cl_2_ and washed with 1M HCl, water, and brine. The organic layer was concentrated in vacuo and purified with column chromatography to obtain **11** (438 mg, 97%) as a colorless oil (see Supplementary Materials, p. 9). ${\left[\alpha \right]}_{D}^{16}$ −12.2 (*c* 1, CHCl_3_). R_f_ = 0.19 (toluene:EtOAc 4:1). ^1^H NMR (300 MHz, CDCl_3_) δ 1.37 and 1.45 (both s, 6H, *Me*_2_C), 1.88, 1.98, and 2.05 (each s, 9H, -C(O)OC*H*_3_), 2.12–2.21 (m, 1H, OH), 3.50 (d, 2H, *J_5,6_* = 4.1 Hz, H-6′ab), 3.59–3.67 (m, 1H, H-5’), 3.73–3.83 (m, 1H, H-5b), 3.74–3.93 (m, 1H, H-5a), 4.00–4.07 (m, 1H, H-4), 4.27–4.34 (m, 1H, H-3), 4.41 and 4.49 (AB system, *J_gem_* = 11.9 Hz, -C*H*_2_Ph), 4.52–4.61 (m, 2H, H-1b, H-2), 4.66–4.75 (m, 1H, H-1a), 4.77 (d, 1H, *J*_1,2_ = 8.0 Hz, H-1′), 4.94 (dd, 1H, *J*_1,2_ = 8.0 Hz, *J*_2,3_ = 9.4 Hz, H-2′), 5.05 (t, 1H, *J*_3,4_ = *J*_4,5_ = 9.5 Hz, H-4′), 5.17 (t, 1H, *J*_2,3_ = *J*_3,4_ = 9.4 Hz, H-3′), 7.05–8.10 (m, 10H, Ph). ^13^C NMR (75 MHz, CDCl_3_) δ 20.60, 20.64, and 20.70 (-C(O)O*C*H_3_), 25.5 and 27.5 (*Me*_2_C), 62.4 (C-5), 63.9 (C-1), 68.7 (C-6′), 69.0 (C-4′), 71.7 (C-2′), 73.0 (C-3′), 73.4 (C-5’), 73.6 (-*C*H_2_Ph), 75.4, 75.5, and 75.6 (C-2, C-3, C-4), 98.2 (C-1′), 109.2 (Me_2_*C*), 125.8, 127.7, 127.9, 128.3, 128.4, 129.7, 130.2, 133.0, 136.5, and 137.7 (Ph), 166.3 (Ph*C*(O)O-), 169.3, 169.4, and 170.4 (-*C*(O)OCH_3_). HRMS (ESI): calcd *m*/*z* for [M + Na]^+^ C_34_H_42_O_14_ 697.2467, found 697.2471.

#### 3.2.5. [1-*O*-Benzoyl-4-*O*-(6-*O*-benzyl-2,3,4-tri-*O*-acetyl-β-d-glucopyrano-syl-2,3-*O*-isopropylidene)-d-ribitol]-5-yl Hydrogenphosphonate Triethylammonium Salt (**12**)

To a mixture of H_3_PO_3_ (586 mg, 7.15 mmol) and PivCl (398 μL, 3.25 mmol) in Py (5 mL), primary alcohol **11** (438 mg, 0.65 mmol) was added. The reaction mixture was stirred at 90 °C for 1 h, diluted with CH_2_Cl_2_, washed with 1M HCl, water, and brine, and then concentrated in vacuo and purified with column chromatography to provide H-phosphonate **12** (324 mg, 74%) as a white amorphous powder (see Supplementary Materials, p. 11). R_f_ = 0.49 (EA:acetone:CH_2_Cl_2_:MeOH:H_2_O 20:15:6:5:4). ^1^H NMR (300 MHz, CDCl_3_) δ 1.35 and 1.45 (both s, 6H, *Me*_2_C), 1.85, 1.95, and 2.05 (each s, 9H, -C(O)OC*H*_3_), 3.55 (d, 2H, *J*_5,6_ = 3.2 Hz, H-6′ab), 3.63–3.76 (m, 1H, H-5′), 3.90–4.05 (m, 2H, H-5ab), 4.05–4.36 (m, 2H, H-4, H-3), 4.42 and 4.49 (AB system, *J_gem_* = 12.0 Hz, -C*H*_2_Ph), 4.52–4.58 (m, 1H, H-2), 4.62 (d, 1H, *J*_1,2_ = 7.4 Hz, H-1b), 4.74 (d, 1H, *J*_1,2_ = 10.9 Hz, H-1a), 4.88–5.13 (m, 3H, H-1′, H-2′, H-4′), 5.13–5.32 (m, 1H, H-3′), 5.80 (s, ½ H, H-P), 7.05–8.15 (m, 10H, Ph), 7.85 (s, ½ H, H-P). ^13^C NMR (75 MHz, CDCl_3_) δ 20.6, 20.7, and 21.0 (-OC(O)*C*H_3_), 25.6 and 27.7 (*Me*_2_C), 64.1 (C-1), 66.8 (C-5), 68.8 (C-6′), 69.3 (C-4′), 71.7 (C-2′), 73.0 (C-3′), 73.4 (C-5′), 74.6 (-*C*H_2_Ph), 75.4 (C-3, C-4), 76.6 (C-2), 98.2 (C-1′), 108.9 (Me_2_*C*), 127.6, 127.8, 128.2, 128.3, 129.8, 130.2, 132.9, and 137.8 (Ph), 166.2 (Ph*C*(O)O-), 166.4, 169.4, and 170.3 (-O*C*(O)CH_3_). ^31^P (122 MHz, CDCl_3_) δ 5.08 (d, *J*_P,H_ = 616.4 Hz). HRMS (ESI): calcd *m*/*z* for [M + H]^+^ C_34_H_43_O_16_P 737.2216, found 737.2211.

#### 3.2.6. (2-(2-(Benzyloxycarbonylamino)ethoxy)ethyl [1-*O*-benzoyl-4-*O*-(6-*O*-benzyl-2,3,4-tri-*O*-acetyl-β-d-glucopyranosyl)-2,3-*O*-isopropylidene-d-ribitol]-5-yl Phosphate Triethylammonium Salt (**13**)

To a solution of H-phosphonate **12** (100 mg, 0.15 mmol) and benzyl (2-(2-hydrohyethohy)ethyl)carbamate (81 mg, 0.30 mmol) in Py (2 mL), PivCl (55 μL, 0.45 mmol) and Et_3_N (42 μL, 0.30 mmol) were added. The reaction mixture was stirred for 15 min at rt until the starting material disappeared (TLC control), and I_2_ (56 mg, 0.22 mmol) and water (100 μL, 5.56 mmol) were then added. The reaction mixture was stirred for 15 min at rt, and it was then quenched with a 0.1N aqueous solution of Na_2_S_2_O_3_, diluted with CH_2_Cl_2_, and washed with 1M HCl, water, and brine. The organic layer was concentrated in vacuo and column chromatography provided **13** (43 mg, 34%) as a white amorphous powder (see Supplementary Materials, p. 14). R_f_ = 0.39 (EA:acetone:CH_2_Cl_2_:MeOH:H_2_O 20:15:6:5:4). ^1^H NMR (300 MHz, CDCl_3_) δ 1.05 (t, 9 H, *J* = 7.4 Hz, (C*H*_3_CH_2_)_3_N), 1.20–1.25 (m, 2H, -NHC*H*_2_CH_2_OCH_2_CH_2_O-), 1.28 and 1.39 (both s, 6H, *Me*_2_C), 1.83, 1.94, and 2.05 (each s, 9H, -C(O)OC*H*_3_), 3.27–3.43 (m, 2H, -NHCH_2_C*H*_2_OCH_2_CH_2_O-), 3.43–3.72 (m, 5H, H-5′, H-6′ab, -NHCH_2_CH_2_OC*H*_2_CH_2_O-), 3.73–3.87 (m, 2H, -NHCH_2_CH_2_OCH_2_C*H*_2_O-), 3.88–4.17 (m, 3H, H-4, H-5ab), 4.25 (s, 1H, H-3), 4.34–4.46 (m, 2H, -C*H*_2_Ph), 4.47–4.64 (m, 2H, H-1b, H-2), 4.71 (d, 1H, *J*_1,2_ = 9.9 Hz, H-1a), 4.96–5.13 (m, 4H, H-1′, H-2′, H-4′, PhC*H*_2_OC(O) NH-), 5.18 (t, 1H, *J*_2,3_ = *J*_3,4_ = 9,1 Hz, H-3′), 7.10–8.15 (m, 15H, Ph). ^13^C NMR (75 MHz, CDCl_3_) δ 20.5, 20.6, and 20.7 (-C(O)O*C*H_3_), 25.6 and 27.7 (*Me*_2_C), 29.7 (NH*C*H_2_CH_2_OCH_2_CH_2_O-), 40.9 (NHCH_2_*C*H_2_OCH_2_CH_2_O-), 41.6 (NHCH_2_CH_2_O*C*H_2_CH_2_O-), 64.2 (C-1), 66.6 (C-5), 67.2 (Ph*C*H_2_O*C*(O) NH-), 68.9 (C-6′), 69.5 (C-4′), 69.9 (NHCH_2_CH_2_OCH_2_*C*H_2_O), 70.7 (C-2′), 71.6 (C-3′), 72.8 (C-5′), 73.0 (-CH_2_-Ph), 73.5 and 74.7 (C-3, C-4), 75.4 (C-2), 98.3 (C-1′), 108.9 (Me_2_*C*), 127.6, 127.8, 128.1, 128.2, 128.3, 128.5, 129.8, 130.2, 132.9, and 137.8 (Ph), 156.8 (Ph*C*(O)O-), 169.4, 169.7, and 170.3 (-*C*(O)OCH_3_). ^31^P (122 MHz, CDCl_3_): δ 1.28. HRMS (ESI): calcd *m*/*z* for [M + H]^+^ C_46_H_58_O_20_P 974.3217, found 974.3214.

#### 3.2.7. 2-Aminoethoxyethyl (4-*O*-β-d-glucopyranosyl)-d-ribitol-5)-yl Phosphate Sodium Salt (**1**)

A suspension of **13** (43 mg, 44 μmol) in CF_3_COOH 90% aq. (73 μL, 0.88 mmol) was placed in an ultrasonic bath for 10 min, diluted with EtOAc, and then coevaporated with toluene. The residue was dissolved in MeOH (440 μL), and 1N MeONa in MeOH (44 μL, 44 μmol) was then added. The reaction mixture was stirred at rt for 1 h until the starting material disappeared (TLC control). The reaction was then quenched with excess CH_3_COOH (10 μL, 0.16 mmol) and P(OH)_2_/C (86 mg, 200 mass%) and H_2_O (440 μL) were added. The reaction mixture was stirred under H_2_ atmosphere at rt for 3 h. The mixture was filtered and concentrated in vacuo. Column chromatography of the residue on TSK HW-40 with 0.1 N CH_3_COOH and lyophilization provided the target disaccharide **1** (8 mg, 36%) as a white powder (see Supplementary Materials, p. 24). R_f_ = 0.32 (EtOH:H_2_O 4:1). ^1^H NMR (300 MHz, D_2_O) δ 3.13–3.19 (m, 2H, NH_2_C*H_2_*CH_2_OCH_2_CH_2_O-), 3.21–3.29 (m, 1H, H-5′), 3.30–3.48 (m, 3H, H-2′, H-4′, H-4), 3.52–3.61 (m, 1H, H-5b), 3.62–3.71 (m, 3H, H-1b, NH_2_CH*_2_*C*H*_2_OCH_2_CH_2_O-), 3.72–3.86 (m, 6H, H-1a, H-2, H-3, H-5a, H-6′ab), 3.96–4.03 (m, 2H, NH_2_CH*_2_*CH_2_OC*H*_2_CH_2_O-), 4.04–4.14 (m, 3H, H-3′, NH_2_CH*_2_*CH_2_OCH_2_C*H*_2_O-), 4.59 (d, *J*_1,2_ = 7.9 Hz, H-1′). ^13^C NMR (75 MHz, D_2_O) δ 39.2 (NH_2_*C*H_2_CH_2_OCH_2_CH_2_O-), 60.6 (C-1), 62.5 (C-5), 64.6 (NH_2_CH_2_*C*H_2_OCH_2_CH_2_O-), 65.05 (NH_2_CH_2_CH_2_O*C*H_2_CH_2_O-), 66.4 (C-6′), 69.6 (C-4′), 70.2 (NH_2_CH_2_CH_2_OCH_2_*C*H_2_O-), 71.4 and 71.7 (C-2, C-3), 73.3 (C-5′), 75.6 and 75.8 (C-4, C-2′), 79.0 (C-3′), 102.3 (C-1′). HRMS (ESI): calcd *m*/*z* for [M + H]^+^ C_15_H_32_O_14_P 480.1477, found 480.1479. ^31^P (122 MHz, D_2_O): δ 0.88.

#### 3.2.8. Compound **14**

To a solution of H-phosphonate **12** (100 mg, 0.14 mmol) and alcohol 9 (119 mg, 0.16 mmol) in Py (2 mL), PivCl (51 μL, 0.42 mmol) and Et_3_N (39 μL, 0.28 mmol) were added. The reaction mixture was stirred for 15 min at rt until the starting material disappeared (TLC control), and I_2_ (51 mg, 0.20 mmol) and H_2_O (100 μL, 5.56 mmol) were then added. The reaction mixture was stirred for 15 min at rt, and it was then quenched with a 0.1N aqueous solution of Na_2_S_2_O_3_, diluted with CH_2_Cl_2_, and washed with 1M HCl, water, and brine. The organic layer was concentrated in vacuo and column chromatography provided **14** (160 mg, 77%) as a white amorphous powder (see Supplementary Materials, p. 17). R_f_ = 0.53 (EA:acetone:CH_2_Cl_2_:MeOH:H_2_O 20:15:6:5:4). ^1^H NMR (300 MHz, CDCl_3_) δ 0.07 and 0.08 (both s, 6H, Si(C*H*_3_)_2_), 0.91 (s, 9H, -C(C*H*_3_)_3_), 1.30 and 1.33 (both s, 6H, *Me*_2_C), 1.83, 1.96, 2.03, 2.07, and 2.11 (each s, 15H, -C(O)OC*H*_3_), 3.51 (d, 2H, J*_gem_* = 3.8 Hz, H-6′ab), 4.55–4.64 (m, 1H, C*H*_2_-Ph), 4.98–5.10 (m, 3H, H-1′, H-1‴, H-4′), 7.09–7.32 (m, 12H, Ph), 7.47–7.57 (m, 2H, Ph), 7.98–8.12 (m, 4H, Ph). ^31^P (122 MHz, CDCl_3_): δ-1.21. HRMS (ESI): calcd *m*/*z* for [M + H]^+^ C_72_H_95_O_29_PSi 1481.5382, found 1481.5370.

#### 3.2.9. Compound **15**

Transformation of tetrasaccharide **14** (160 mg, 0.11 mmol) into derivative **15** (133 mg, 90%) was performed similarly to the process described for the preparation of compound **11** from **10**. R_f_ = 0.41 (EA:acetone:CH_2_Cl_2_:MeOH:H_2_O 20:15:6:5:4). HRMS (ESI): calcd *m*/*z* for [M + H]^+^ C_66_H_81_O_29_P 1367.4517, found 1367.4510.

#### 3.2.10. Compound **16**

Transformation of tetrasaccharide **15** (133 mg, 97 μmol) into derivative **16** (133 mg, 96%) was performed similarly to the process described for the preparation of compound **12** from **11** (see Supplementary Materials, p. 20). R_f_ = 0.21 (EA:acetone:CH_2_Cl_2_:MeOH:H_2_O 20:15:6:5:4). ^31^P (122 MHz, CDCl_3_): δ-1.30, 0.56. Calcd *m*/*z* for [M + H]^+^ C_66_H_82_O_31_P_2_ 1431.4243, found 1431.4247.

#### 3.2.11. Compound **17**

Transformation of tetrasaccharide **16** (133 mg, 93 μmol) into derivative **17** (85 mg, 54%) was performed similarly to the process described for the preparation of compound **13** from **12** (see Supplementary Materials, p. 22). R_f_ = 0.32 (EA:acetone:CH_2_Cl_2_:MeOH:H_2_O 20:15:6:5:4). ^31^P (122 MHz, CDCl_3_): δ-1.31, 0.50. Calcd *m*/*z* for [M + H]^+^ C_78_H_97_O_35_P_2_ 833.7585, found 833.7611.

#### 3.2.12. Compound **2**

Transformation of tetrasaccharide **17** (85 mg, 50 μmol) into derivative **2** (25 mg, 59%) was performed similarly to the process described for the preparation of compound **1** from **13** (see Supplementary Materials, p. 27). R_f_ = 0.29 (EtOH-H_2_O 4:1). ^1^H NMR (300 MHz, D_2_O, selected data) δ 3.31–3.34 (m, 2H, NH_2_C*H_2_*CH_2_OCH_2_CH_2_O-), 4.59 (d, 1H, *J*_1,2_ = 7.9 Hz, H-1′), 4.61 (d, 1H, *J*_1,2_ = 7.9 Hz, H-1‴). ^13^C NMR (75 MHz, D_2_O, selected data) δ 39.1 (NH_2_*C*H*_2_*CH_2_OCH_2_CH_2_O-), 102.2 (C-1′), 102.3 (C-1‴). ^31^P (122 MHz, D_2_O) δ 0.42, 0.90. Calcd *m*/*z* for [M + H]^+^ C_26_H_53_O_26_P_2_ 856.2258, found 856.2264.

#### 3.2.13. Compound **18**

To a solution of H-phosphonate **16** (100 mg, 70 μmol) and alcohol **9** (63 mg, 84 μmol) in Py (2 mL), PivCl (26 μL, 0.21 mmol) and Et_3_N (29 μL, 0.21 mmol) were added. The reaction mixture was stirred for 15 min at rt until the starting material disappeared (TLC control), and I_2_ (26 mg, 0.10 mmol) and H_2_O (100 μL, 5.56 mmol) were then added. The reaction mixture was stirred for 15 min at rt, and it was then quenched with 0.1N Na_2_S_2_O_3_, diluted with CH_2_Cl_2_, and washed with 1M HCl, water, and brine. The organic layer was concentrated in vacuo and column chromatography provided **18** (69 mg, 45%) as a white amorphous powder. R_f_ = 0.36 (EA:acetone:CH_2_Cl_2_:MeOH:H_2_O 20:15:6:5:4). Calcd *m*/*z* for [M + H]^+^ C_104_H_134_O_44_P_2_Si 2175.7420, found 2175.7415.

#### 3.2.14. Compound **21**

Compound **18** (69 mg, 31 μmol) was dissolved in a mixture of Py:HF (90% aq.) at a ratio of 6:1 (1 mL) in a plastic centrifuge tube. The reaction mixture was heated to 60 °C and stirred for 2 h until the starting material disappeared (TLC control). It was then diluted with CH_2_Cl_2_ and washed with 1M HCl and brine. The organic layer was concentrated in vacuo. The mixture of H_3_PO_3_ (28 mg, 0.34 mmol) and PivCl (20 μL, 0.16 mmol) in Py (500 μL) was added to the residue. The reaction mixture was stirred at 90 °C for 1 h, diluted with CH_2_Cl_2_, and then washed with 1M HCl, water, and brine. The organic layer was concentrated in vacuo. The residue was dissolved in Py (500 μL), then benzyl (2-(2-hydrohyethohy)ethyl)carbamate (17 mg, 62 μmol), PivCl (11 μL, 90 μmol), and Et_3_N (22 μL, 0.16 mmol) were added. The reaction mixture was stirred for 15 min at rt until the starting material disappeared (TLC control), and I_2_ (12 mg, 47 μmol) and water (25 μL, 1.4 mmol) were then added. The reaction mixture was stirred for 15 min at rt, and it was then quenched with a 0.1 N aqueous solution of Na_2_S_2_O_3_, diluted with CH_2_Cl_2_, and washed with 1M HCl, water, and brine. The organic layer was concentrated in vacuo and column chromatography provided **21** (32 mg, 44%) as a white amorphous powder. R_f_ = 0.15 (EA:acetone:CH_2_Cl_2_:MeOH:H_2_O 20:15:6:5:4). Calcd *m*/*z* for [M + H]^+^ C_110_H_136_O_50_P_3_ 787.2386, found 787.2396.

#### 3.2.15. Compound **3**

Transformation of hexasaccharide **21** (32 mg, 14 μmol) into derivative **3** (15 mg, 90%) was performed similarly to the process described for the preparation of compound **1** from **13** (see Supplementary Materials, p. 30). R_f_ = 0.38 (EtOH:H_2_O 4:1). ^1^H NMR (300 MHz, D_2_O, selected data) δ 3.22–3.25 (m, 2H, NH_2_C*H_2_*CH_2_OCH_2_CH_2_O-), 4.66 (d, 1H, *J*_1,2_ = 8.3 Hz, H-1′), 4.67 (d, 2H, *J*_1,2_ = 8.7 Hz, H-1‴, H-1″‴). ^13^C NMR (75 MHz, D_2_O, selected data) δ 39.2 (NH_2_*C*H*_2_*CH_2_OCH_2_CH_2_O-), 103.6 (C-1′), 103.7 (C-1‴), 103.69 (C-1″‴). ^31^P (122 MHz, D_2_O) δ 0.42, 0.90. HRMS (ESI): calcd *m*/*z* for [M + H]^+^ C_37_H_73_O_38_P_3_ 1232.3029, found 1232.3004.

#### 3.2.16. Compound **4**

To a solution of **3** (1 mg, 0.8 μmol) in DMSO (150 μL), Et_3_N (12 μL, 0.12 mmol) and a 62 μmol/mL solution of compound 22 in DMSO (15 μL, 0.96 μmol) were added. The reaction mixture was incubated for 1 h until the starting material disappeared (TLC control). The target biotin derivative **4** was then purified through column chromatography on the TSK HW-40 with 0.1 N CH_3_COOH, which was then followed by lyophilization to provide **4** (1 mg, 70%) (see Supplementary Materials, p. 33). R_f_ = 0.65 (EtOH:H_2_O 4:1). ^1^H NMR (600 MHz, D_2_O, characteristic signals): oligosaccharide fragment: 4.63 (d, 2 H, *J*_1,2_ = 8.2 Hz, H-1‴, H-1″‴), 4.64 (d, 1 H, *J*_1,2_ = 8.4 Hz, H-1′); biotin fragment: 1.32–1.72 (m, 6H, H-3, H-4, H-5), 2.23 (t, 2H, *J* = 7.3 Hz, H-2), 2.50 (t, 2 H, *J* = 6.0, C(O)C*H*_2_CH_2_), 2.73 (d, 1H, *J* = 13.1 Hz, H-6_cis_), 2.94 (dd, 1H, *J* = 4.8, 13.1 Hz; H-6_trans_), 4.38 (dd, 1H, *J* = 5.0 Hz; 7.9 Hz; H-3a), 4.56 (dd, 1 H, *J* = 5.0 Hz; 7.9 Hz; H-6a). HRMS (ESI): calcd *m*/*z* for [M − 2H]/2^2−^ C_62_H_117_O_47_N_4_P_3_S 896.2833, found 896.2908.

## 4. Conclusions

Initial synthesis of spacer-armed pseudodi- (**1**), pseudotetra- (**2**), and pseudohexasaccharide (**3**) derivatives related to the *Hia* CG was performed efficiently using the convergent chain elongation scheme and H-phosphonate chemistry. Compounds **1**–**3** were obtained for application as haptens in inhibitory ELISA and conjugation with immune-tolerable protein carriers in the design of glycoconjugate vaccines. The biotinylated conjugate **4** will be used as a coating antigen in ELISA testing for immobilization on streptavidin-coated plates and chip slides. The results of immunological studies of compounds **1**–**4** will be published elsewhere.

## Data Availability

Data are available from the corresponding author.

## Supplementary Materials

The following supporting information can be downloaded at: https://www.mdpi.com/article/10.3390/molecules28155688/s1, Copies of the NMR spectra of compounds **7**, **8**, **9**, **10**, **11**, **12**, **13**, **14**, **16**, **17**, **1**, **2**, **3**, and **4**.

## Sample Availability

Samples of described compounds are available from the corresponding author.
