# Supplementary material for: Synthesis of Pseudooligosaccharides Related to the Capsular Phosphoglycan of Haemophilus influenzae Type a
Source: Molecules. 2023 Jul 27;28(15):5688. doi: 10.3390/molecules28155688 (PMC10419796; doi:10.3390/molecules28155688)
Supplement: Supplementary file 1 [file molecules-28-05688-s001.zip › molecules-2525165-supplementary.pdf]

Supplementary Materials

Synthesis of Pseudooligosaccharides Related to the Capsular Phosphoglycan of *Haemophilus influenzae* Type *a*

Anastasia A. Kamneva, Dmitry V. Yashunsky, Elena A. Khatuntseva and Nikolay E. Nifantiev *

N. D. Zelinsky Institute of Organic Chemistry, Leninsky pr. 47, Moscow 119991, Russia.

** Corresponding author. Fax: +7 (499) 135 8784.*

E-mail: nen@ioc.ac.ru (N.E.N.)

Table of Contents

1. ^{1}H and ^{13}C NMR Spectra of compound 7 3
2. ^{1}H and ^{13}C NMR Spectra of compound 8 5
3. ^{1}H and ^{13}C NMR Spectra of compound 10 7
4. ^{1}H and ^{13}C NMR Spectra of compound 11 9
5. ^{1}H, ^{13}C and ^{31}P NMR Spectra of compound 12 11
6. ^{1}H, ^{13}C and ^{31}P NMR Spectra of compound 13 14
7. ^{1}H, ^{13}C and ^{31}P NMR Spectra of compound 14 17
8. ^{1}H and ^{31}P NMR Spectra of compound 16 20
9. ^{1}H and ^{31}P NMR Spectra of compound 17 22
10. ^{1}H, ^{13}C and ^{31}P NMR Spectra of compound 1 24
11. ^{1}H, ^{13}C and ^{31}P NMR Spectra of compound 2 27
12. ^{1}H, ^{13}C and ^{31}P NMR Spectra of compound 3 30
13. ^{1}H NMR Spectrum of compound 4 33

1. ^{1}H and ^{13}C NMR Spectra of compound 7

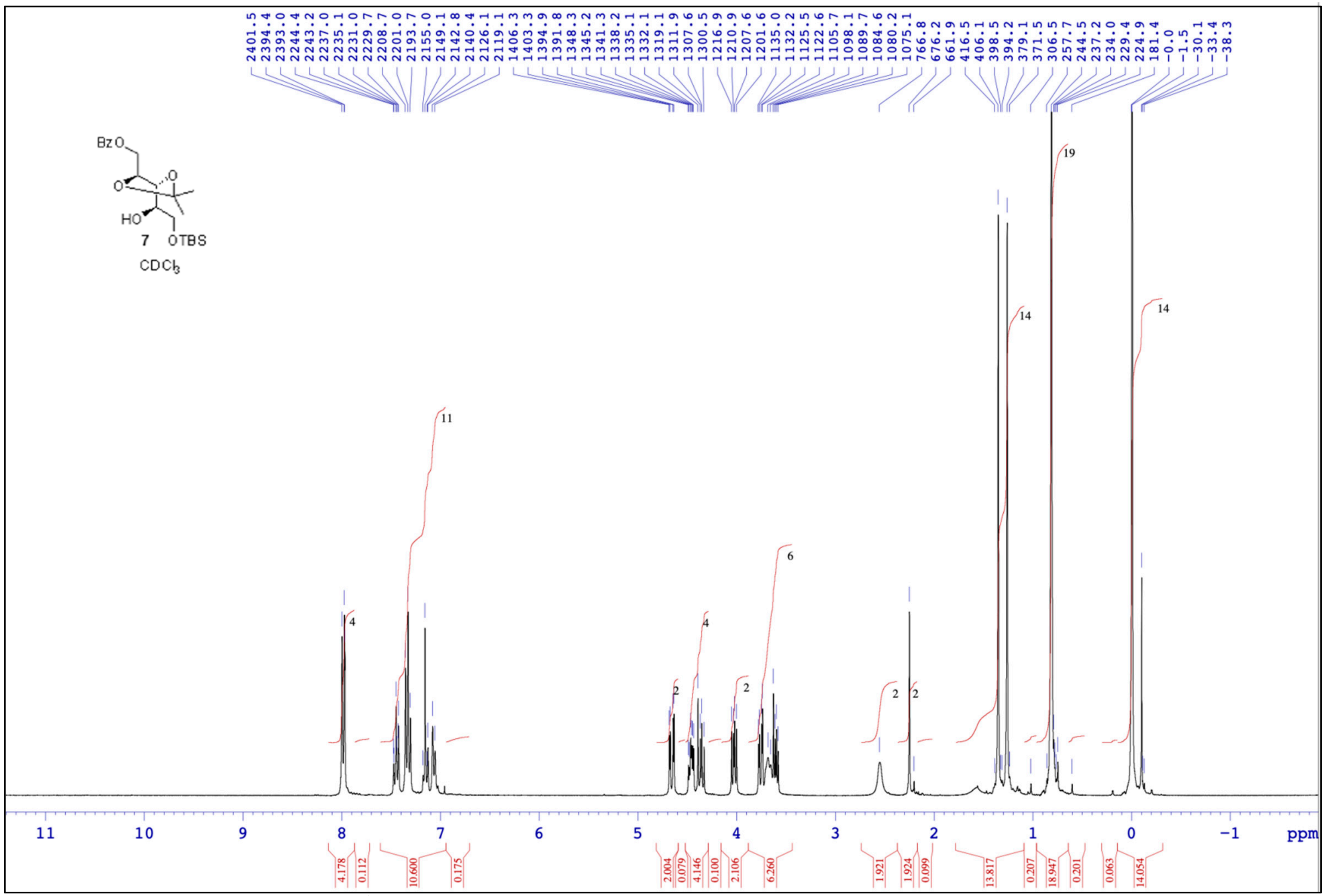

^{1}H NMR spectrum of compound **7** (300 MHz, $CDCl_3$)

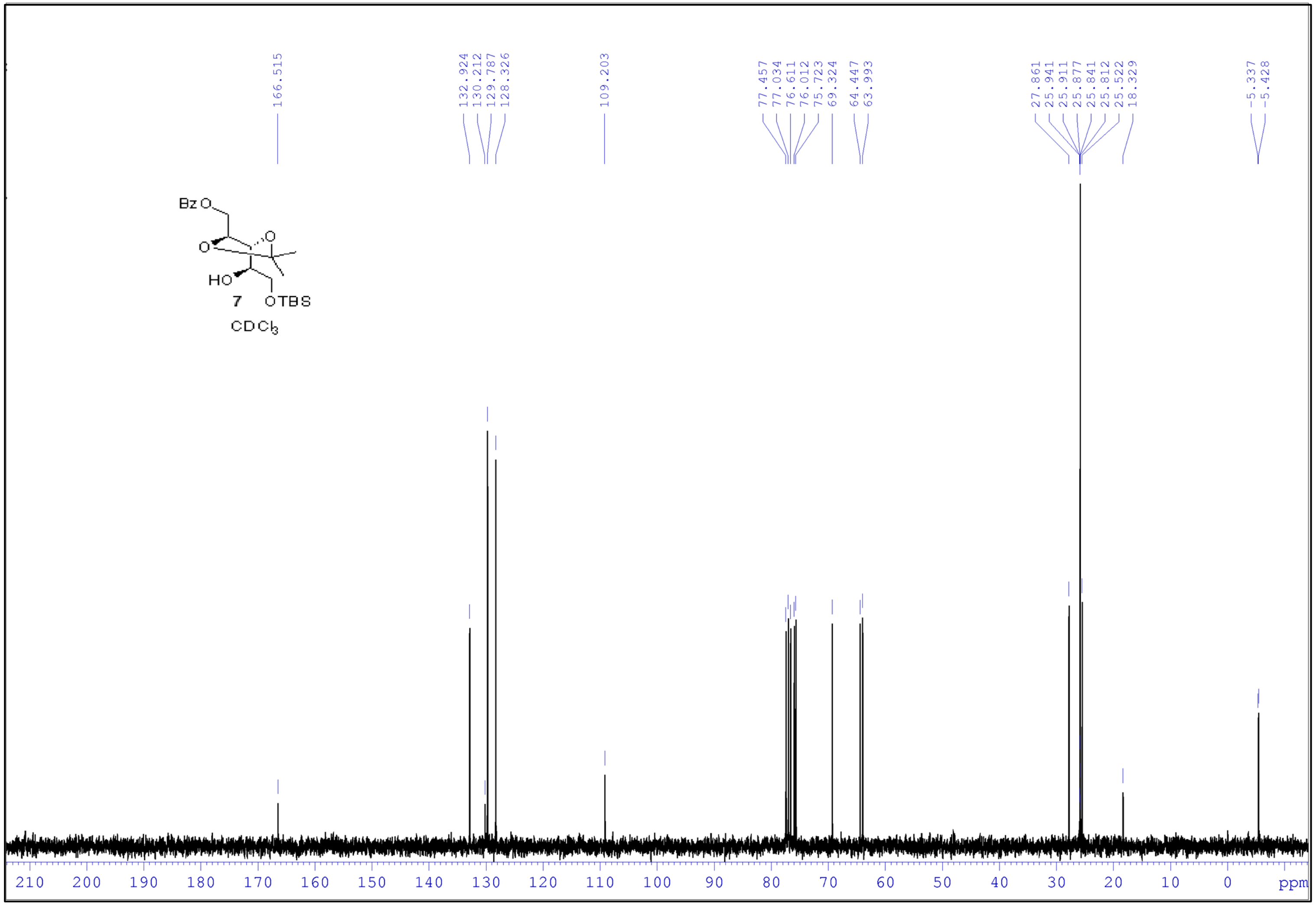

^{13}C NMR spectrum of compound **7** (75 MHz, $CDCl_3$)

2. ^{1}H and ^{13}C NMR Spectra of compound 8

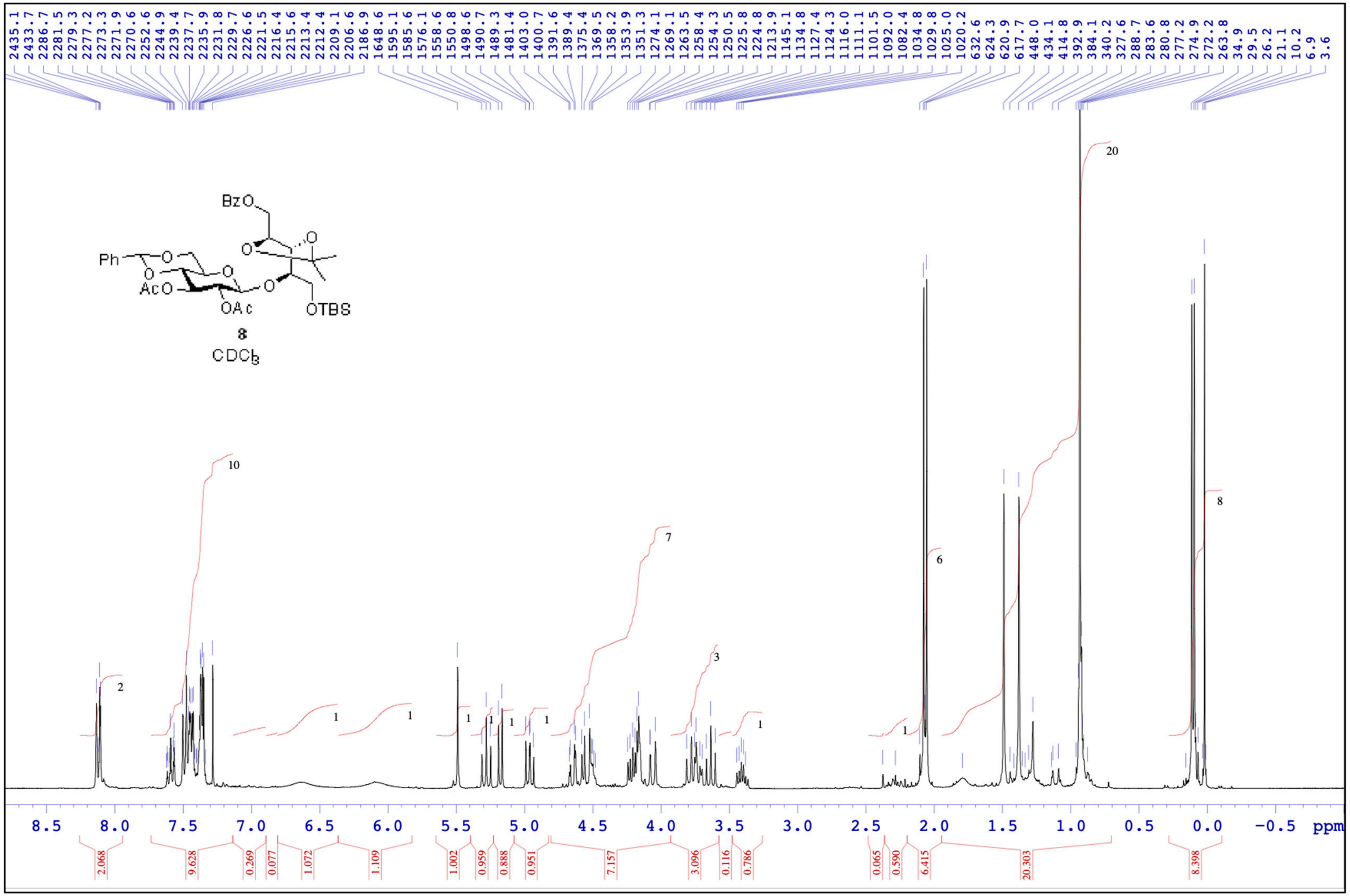

^{1}H NMR spectrum of compound **8** (300 MHz, $CDCl_3$)

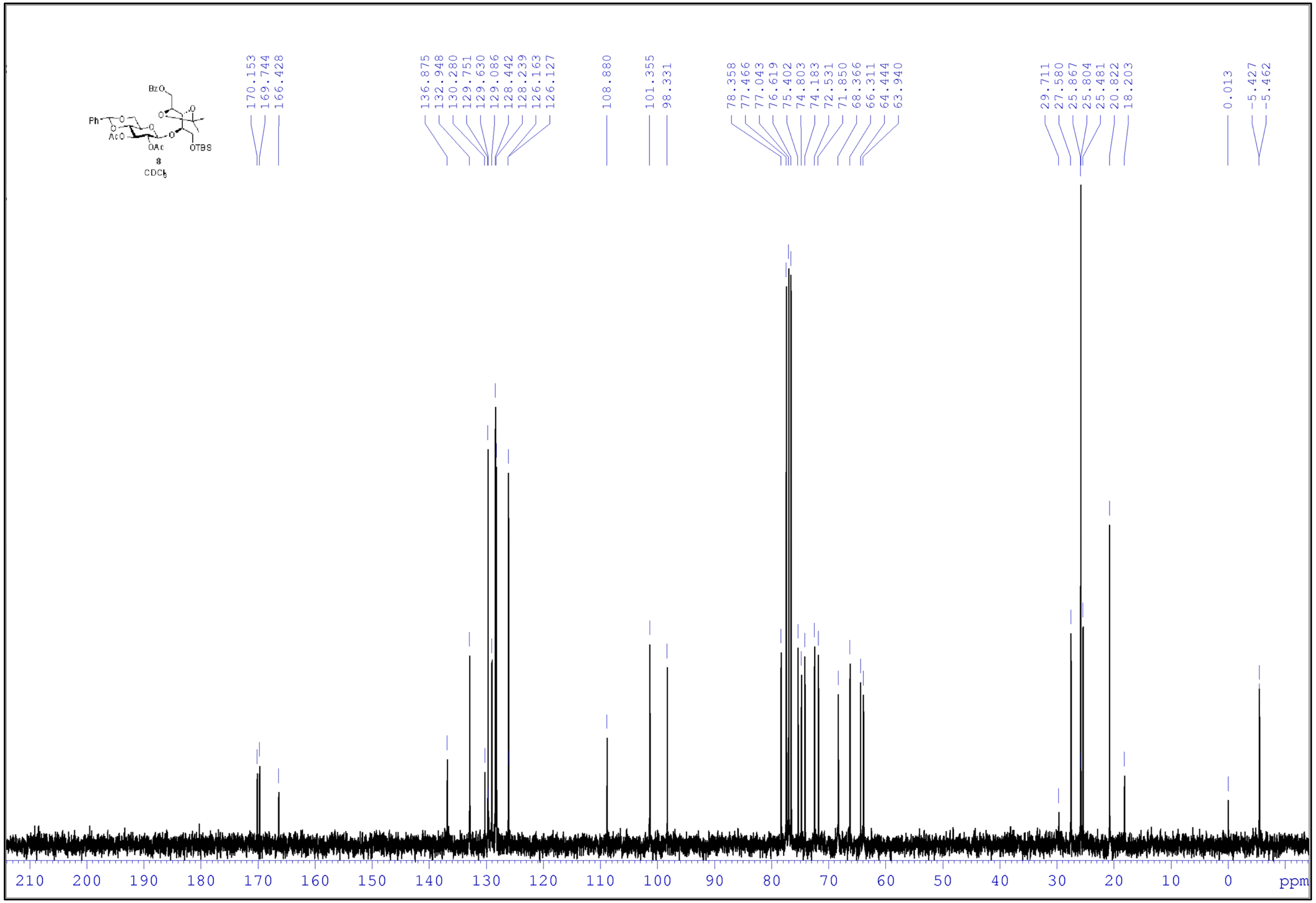

^{13}C NMR spectrum of compound **8** (75 MHz, $CDCl_3$)

3. ¹H and ¹³C NMR Spectra of compound 9

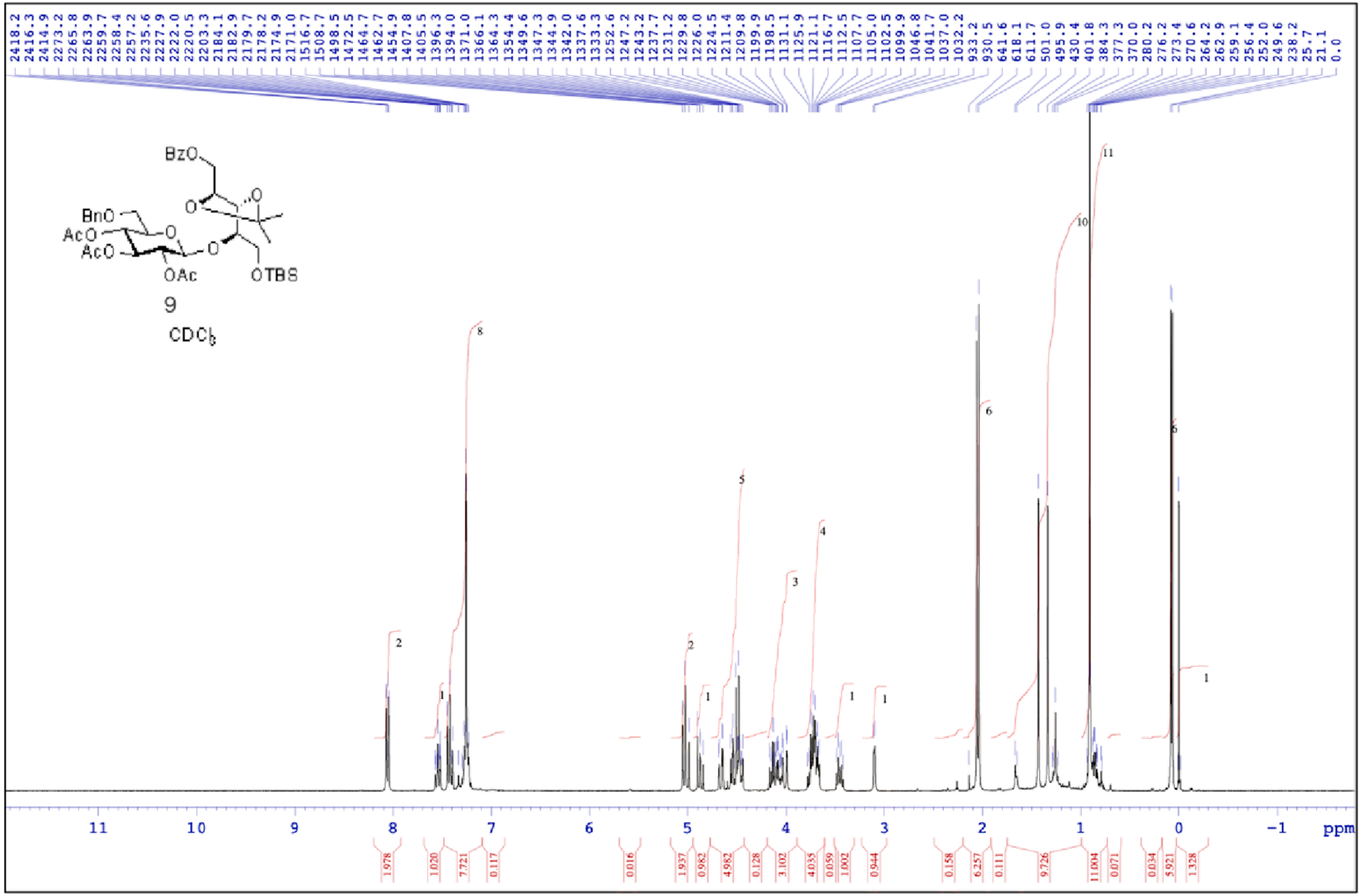

¹H NMR spectrum of compound **9** (300 MHz, $CDCl_3$)

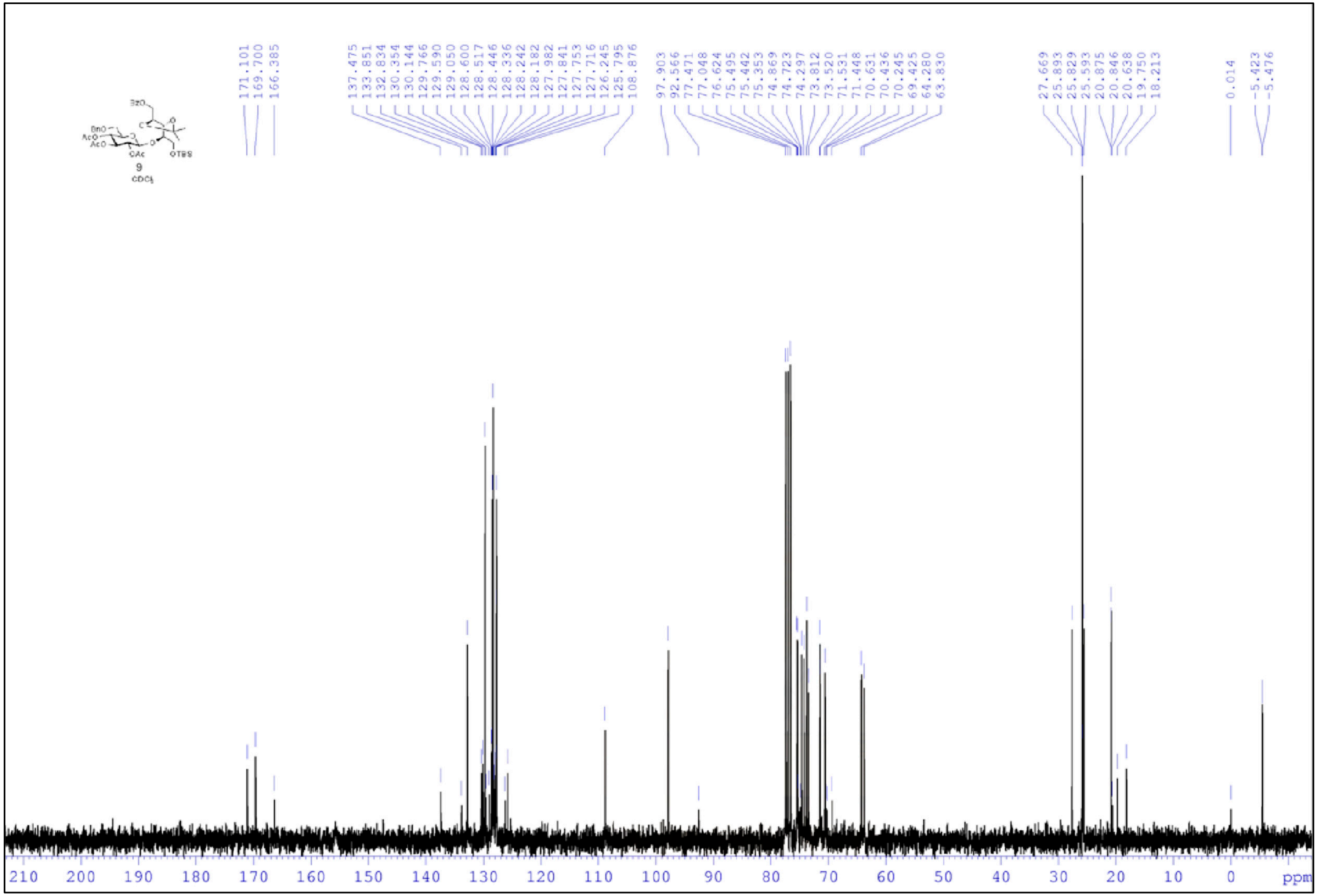

^{13}C NMR spectrum of compound **9** (75 MHz, $CDCl_3$)

4. ^{1}H and ^{13}C NMR Spectra of compound 11

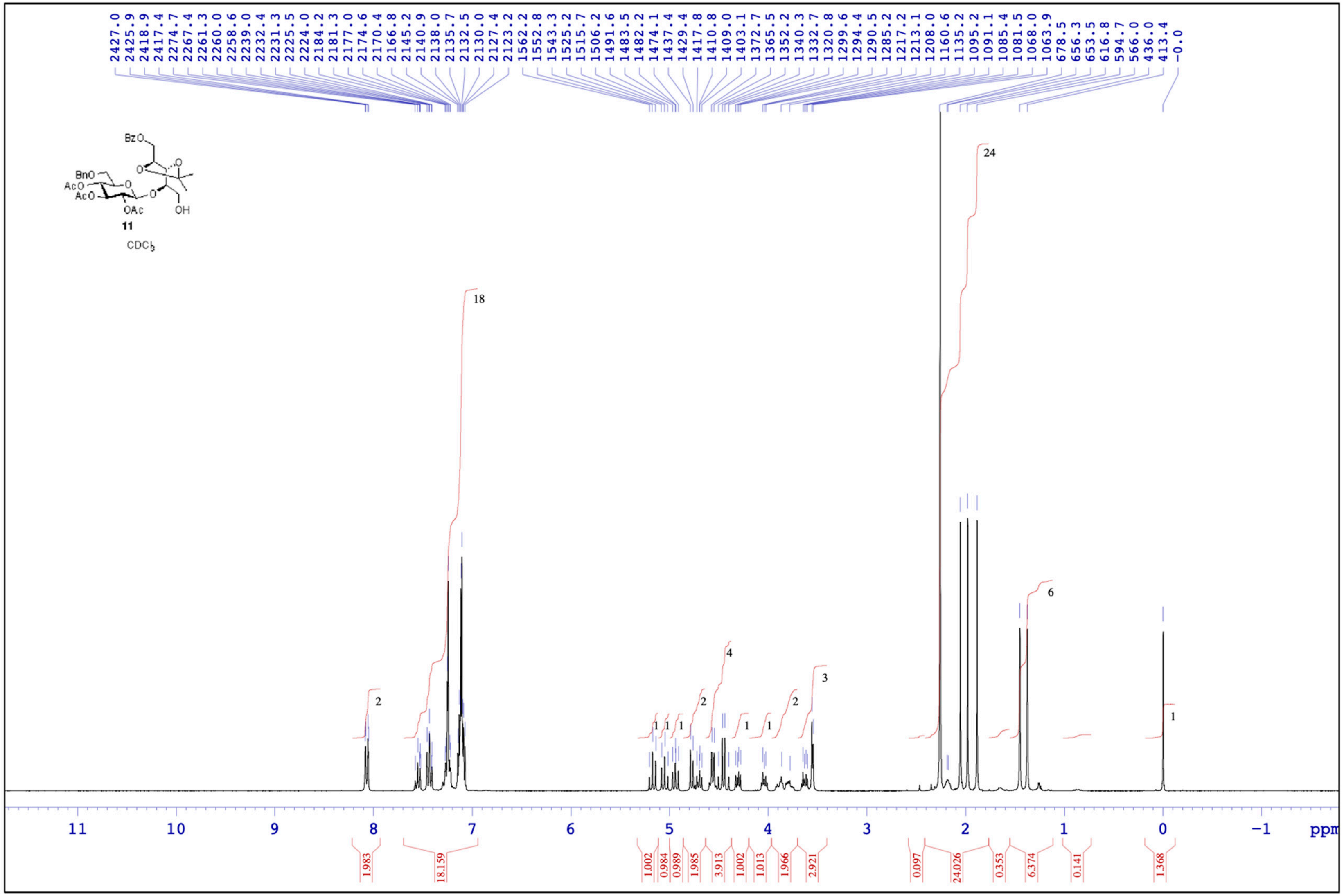

^{1}H NMR spectrum of compound **11** (300 MHz, $CDCl_3$)

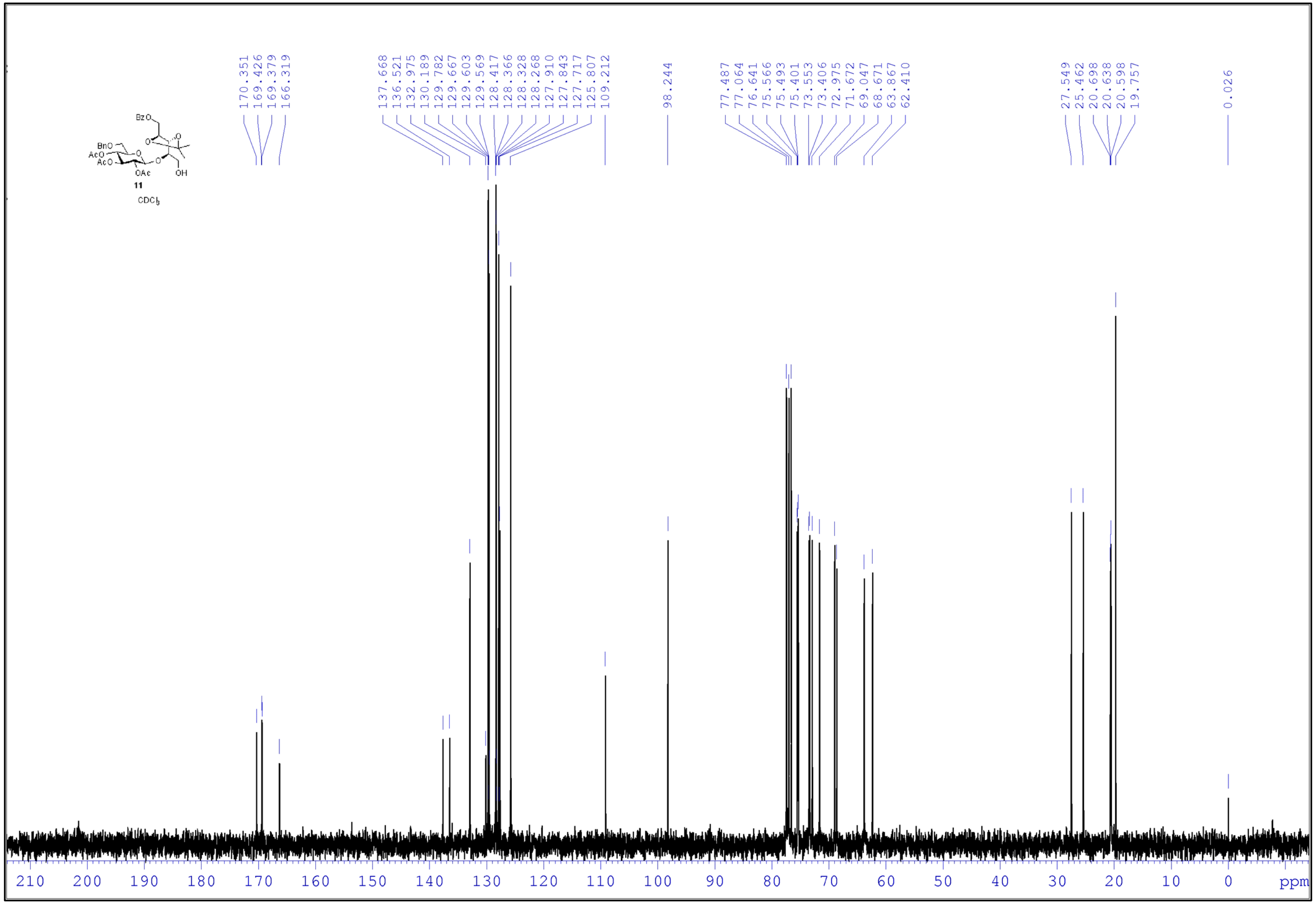

^{13}C NMR spectrum of compound **11** (75 MHz, $CDCl_3$)

5. ^{1}H, ^{13}C and ^{31}P NMR Spectra of compound 12

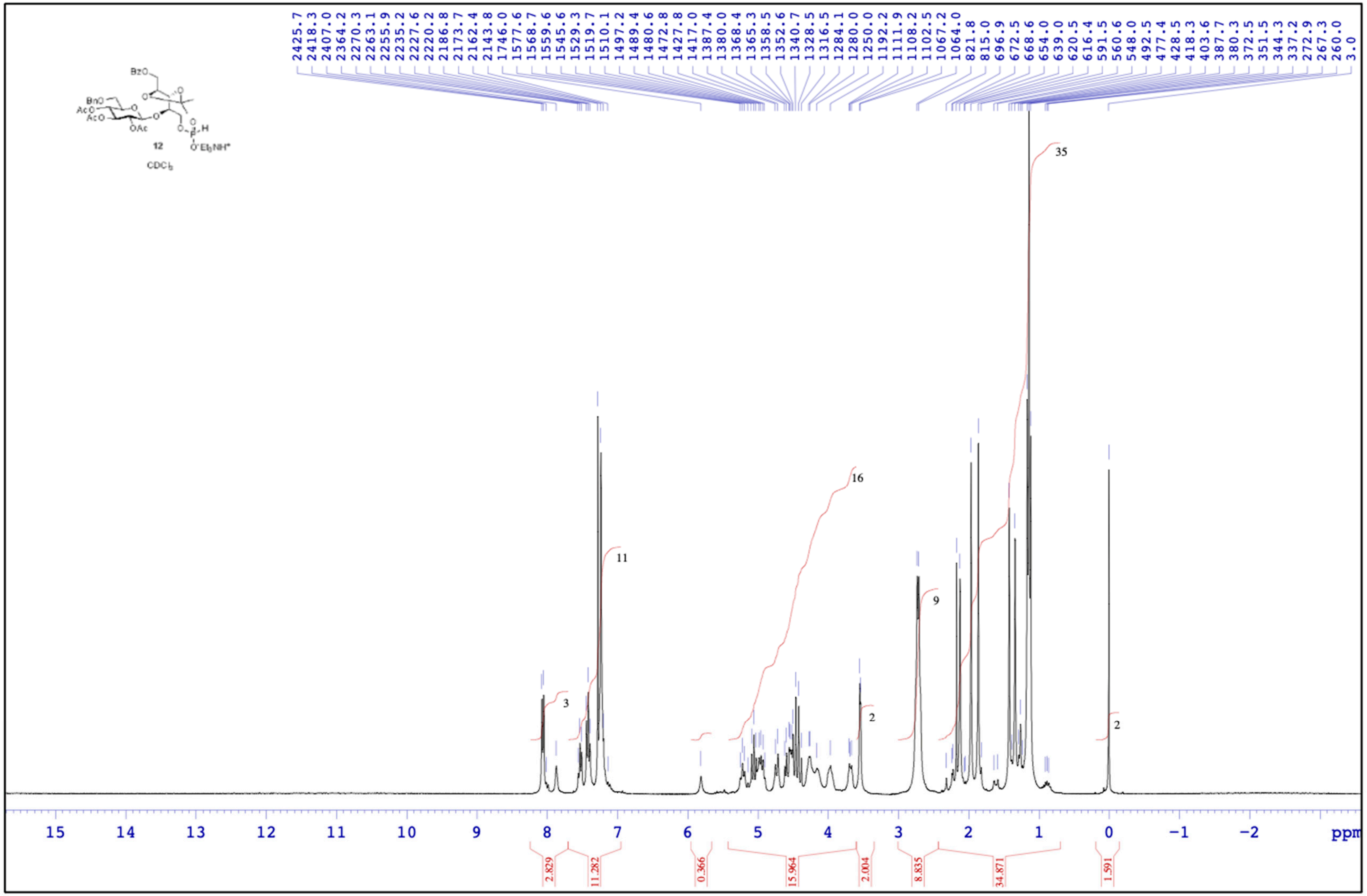

^{1}H NMR spectrum of compound **12** (300 MHz, $CDCl_3$)

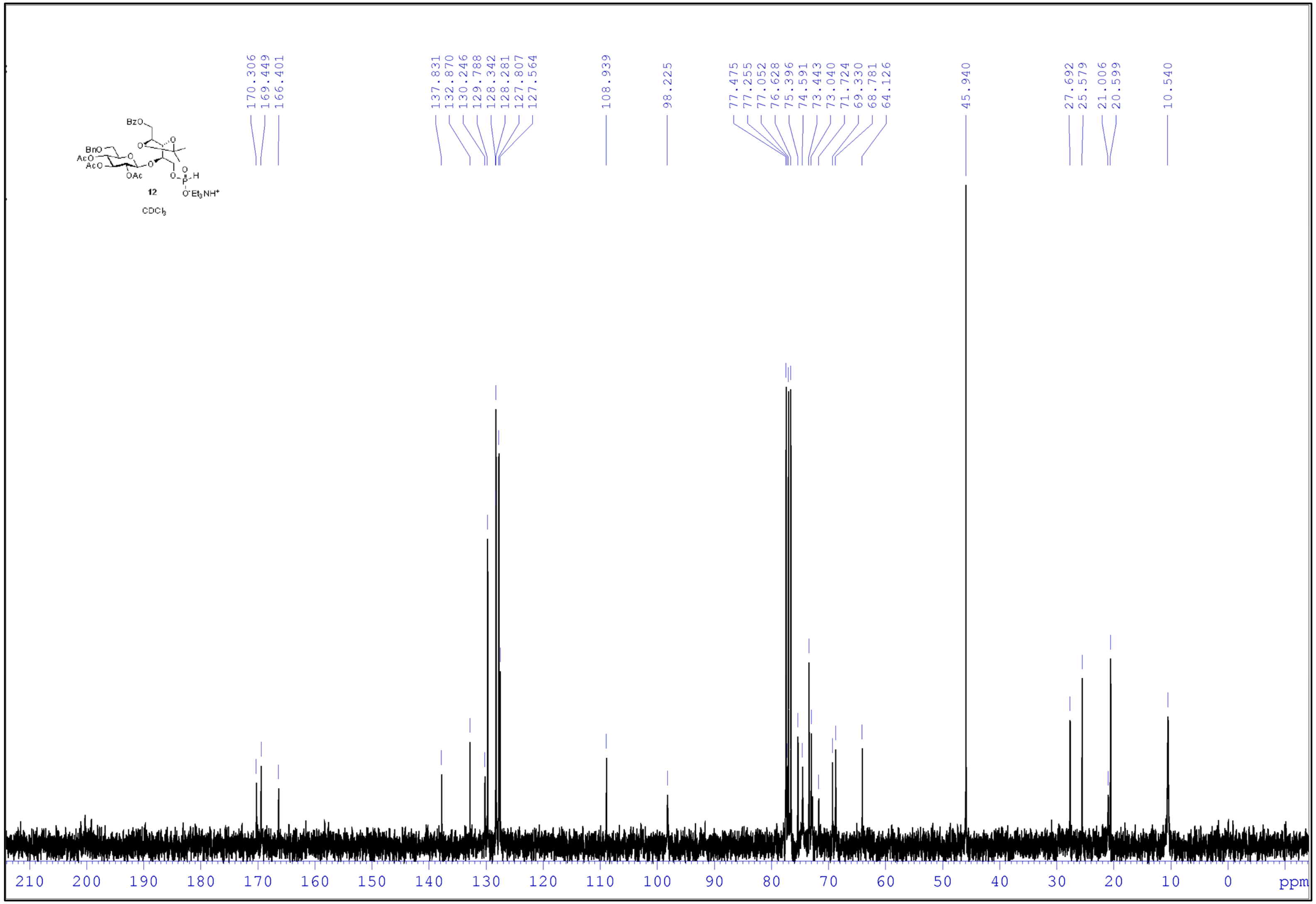

^{13}C NMR spectrum of compound **12** (75 MHz, $CDCl_3$)

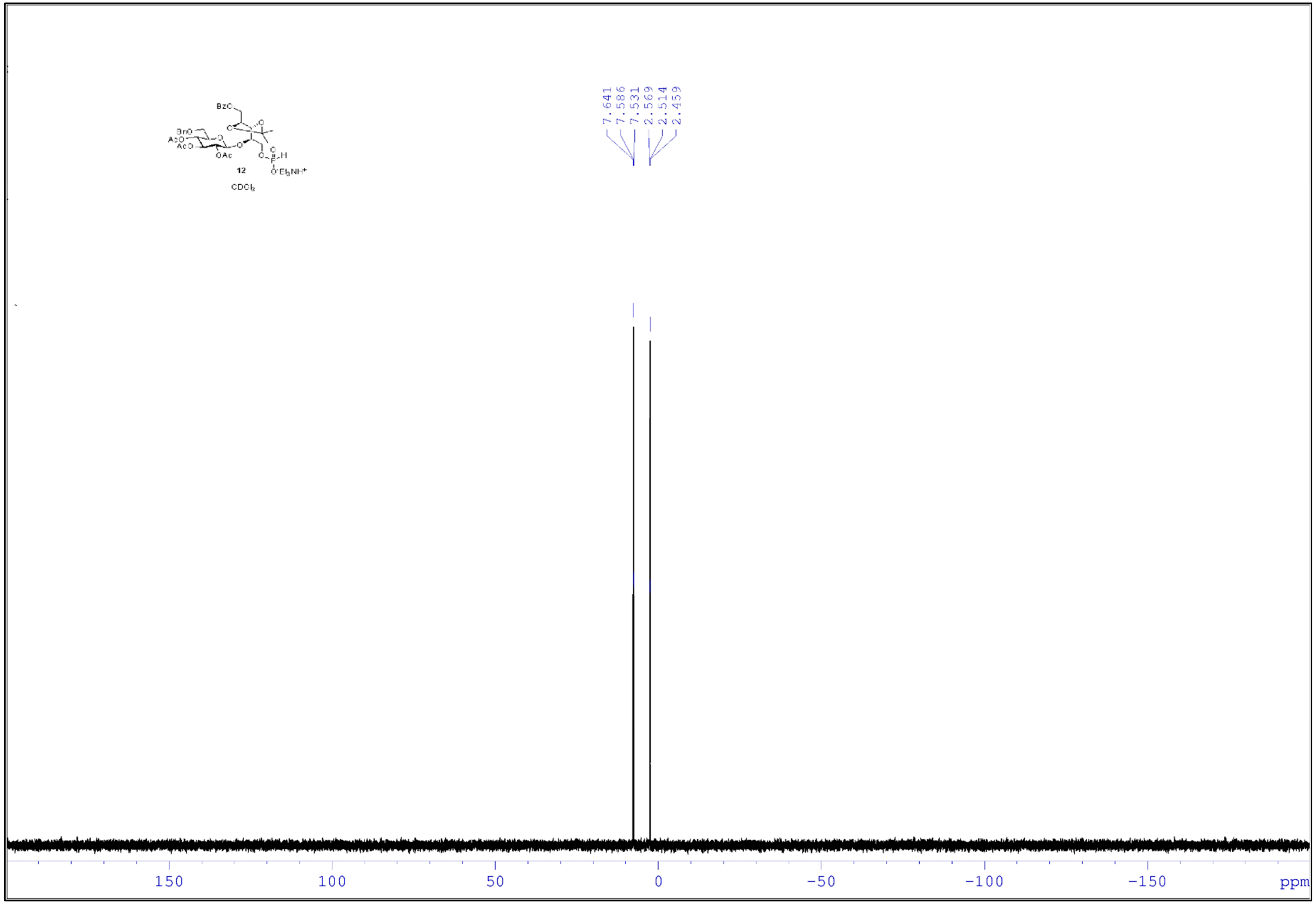

^{31}P NMR spectrum of compound **12** (122 MHz, $CDCl_3$)

6. ^{1}H, ^{13}C and ^{31}P NMR Spectra of compound 13

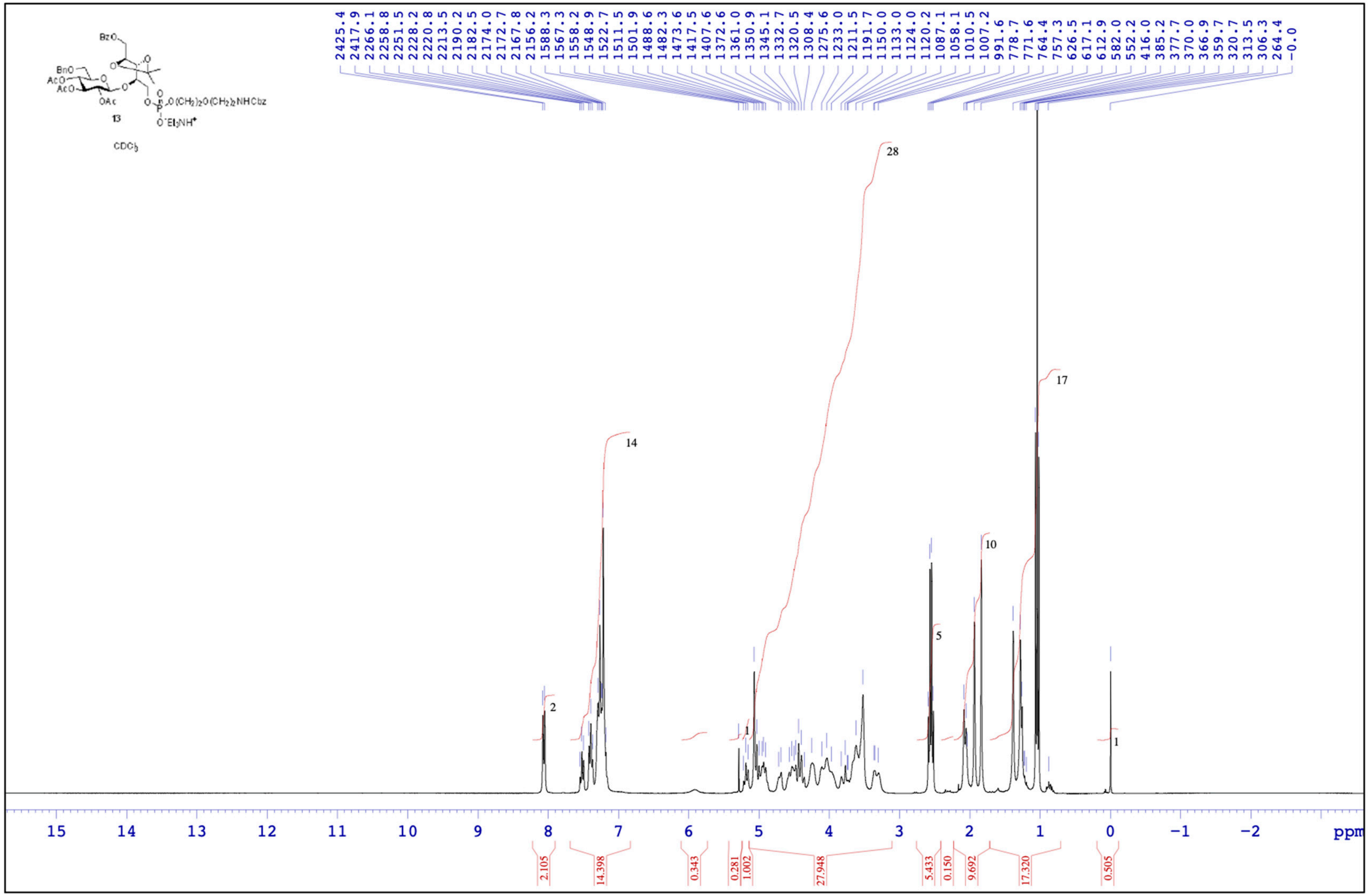

^{1}H NMR spectrum of compound **13** (300 MHz, $CDCl_3$)

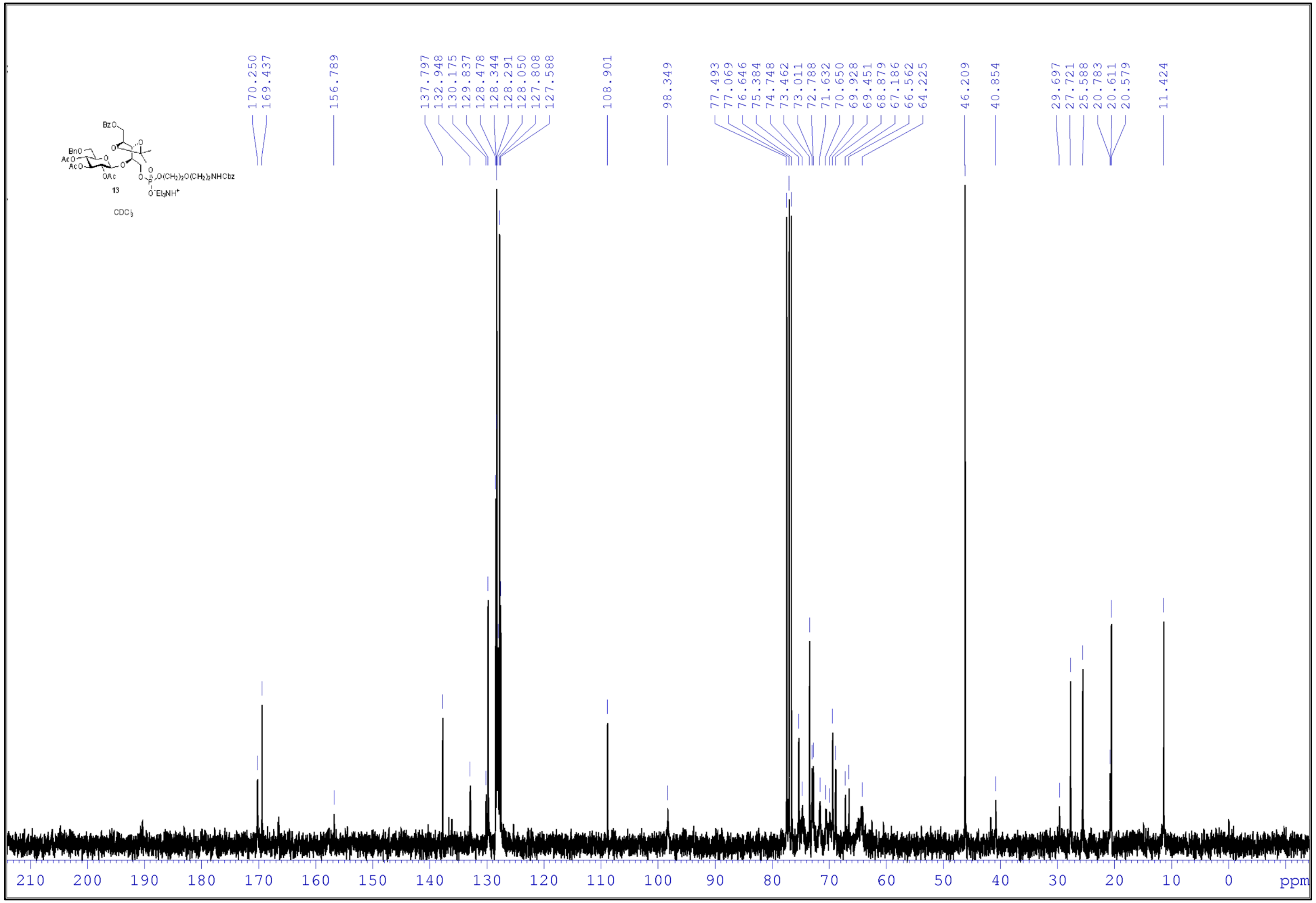

^{13}C NMR spectrum of compound **13** (75 MHz, $CDCl_3$)

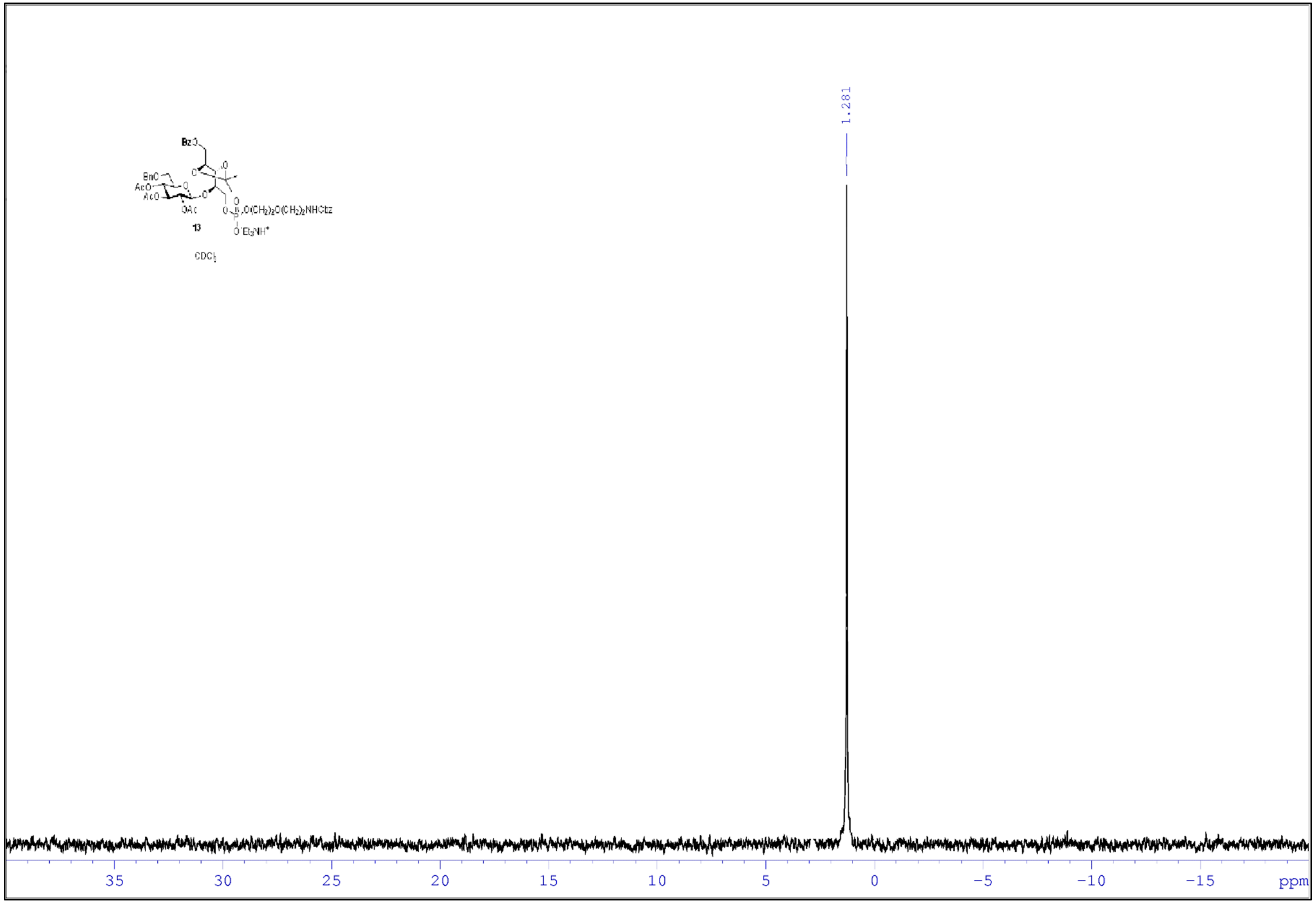

^{31}P NMR spectrum of compound **13** (122 MHz, $CDCl_3$)

7. ^{1}H, ^{13}C and ^{31}P NMR Spectra of compound 14

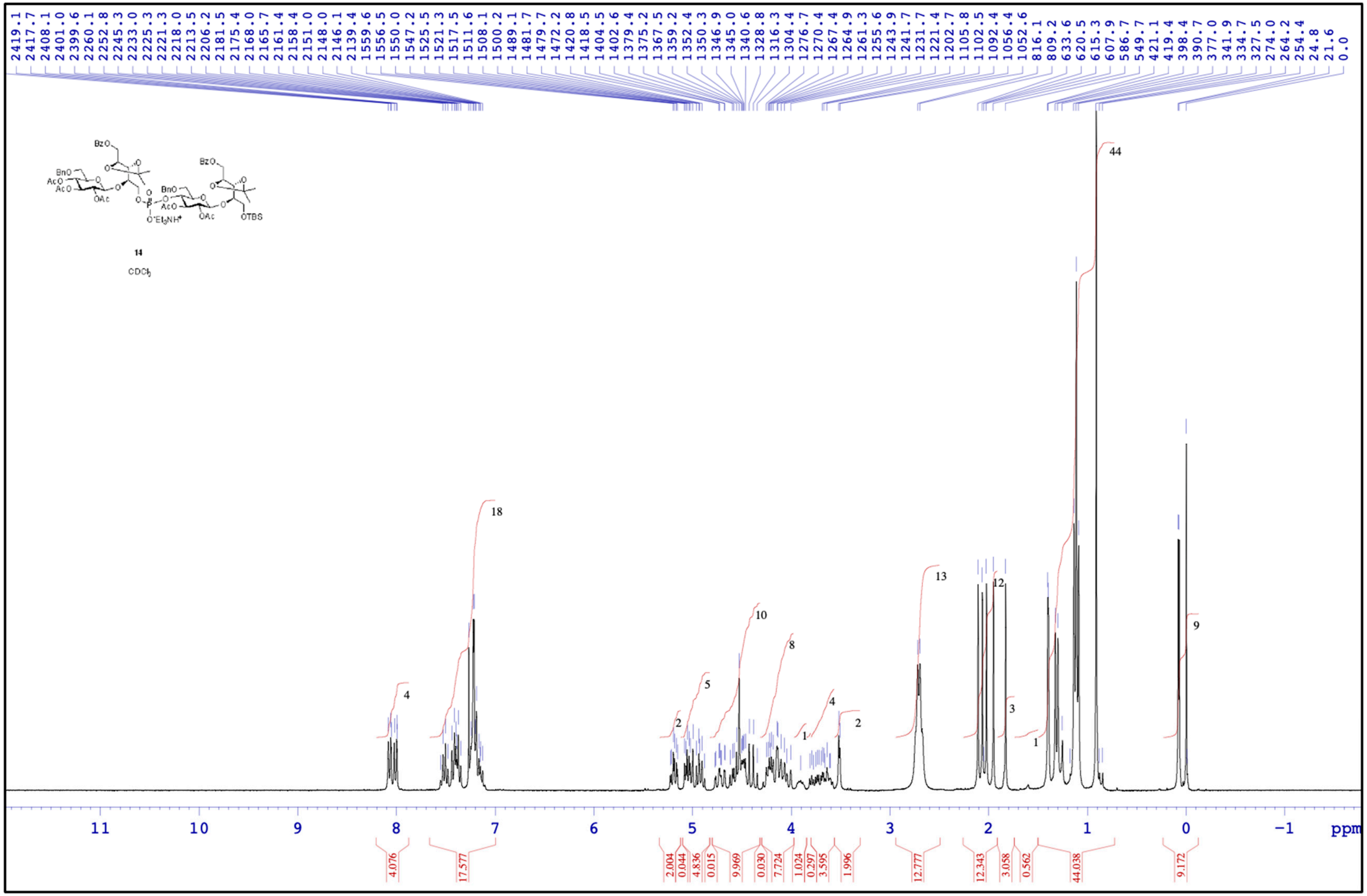

^{1}H NMR spectrum of compound **14** (300 MHz, $CDCl_3$)

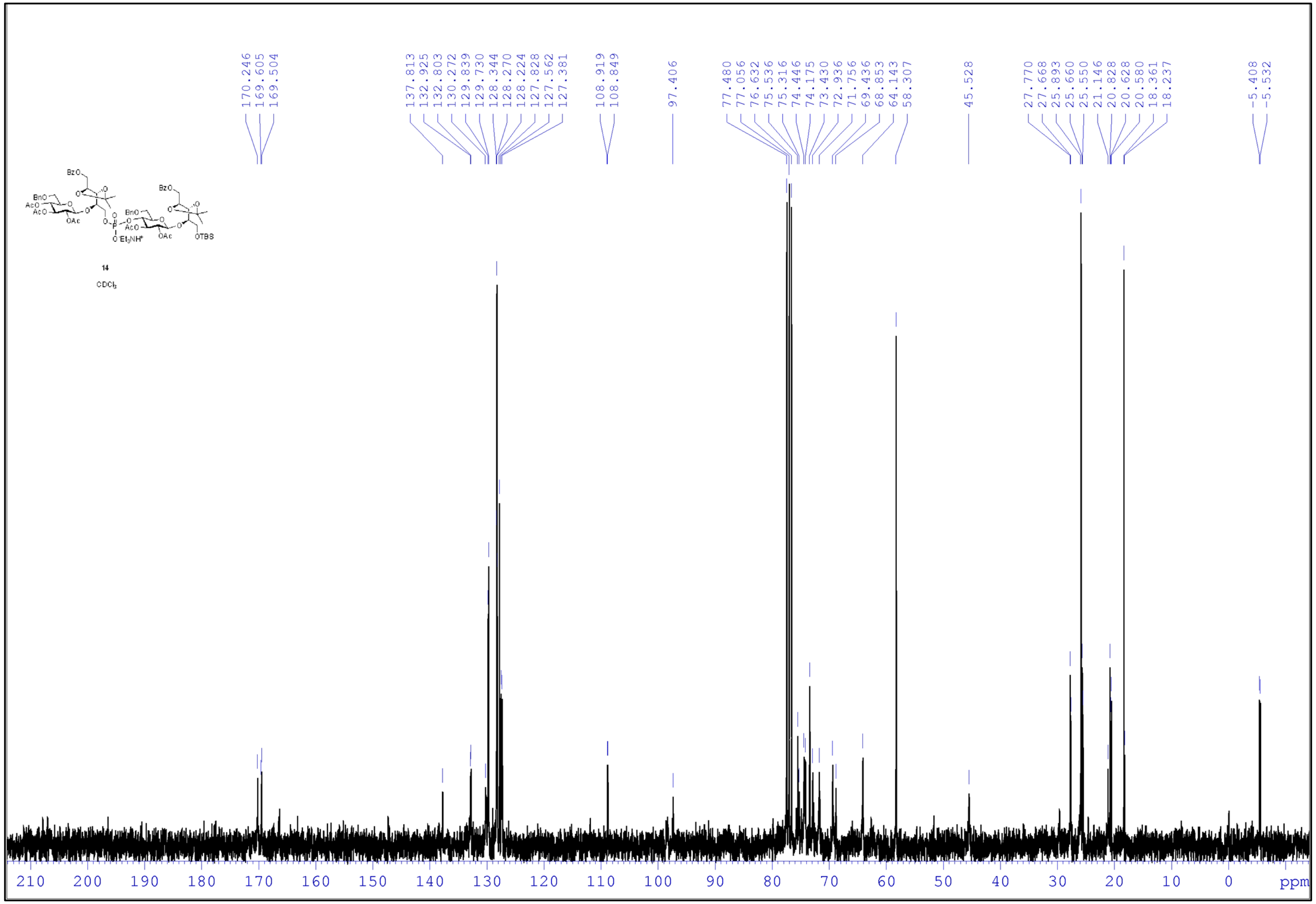

^{13}C NMR spectrum of compound **14** (75 MHz, $CDCl_3$)

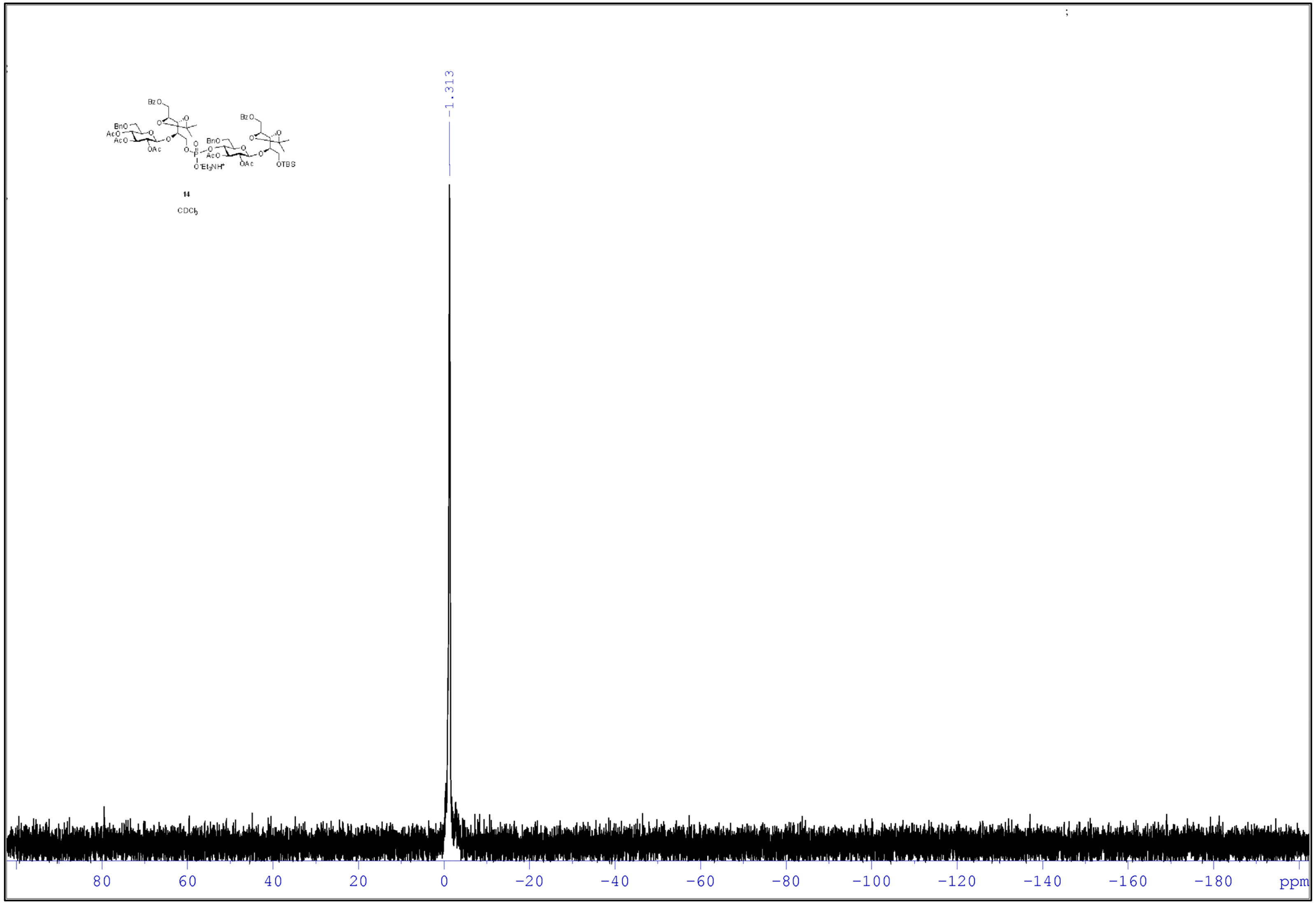

^{31}P NMR spectrum of compound **14** (122 MHz, $CDCl_3$)

8. ^{1}H and ^{31}P NMR Spectra of compound 16

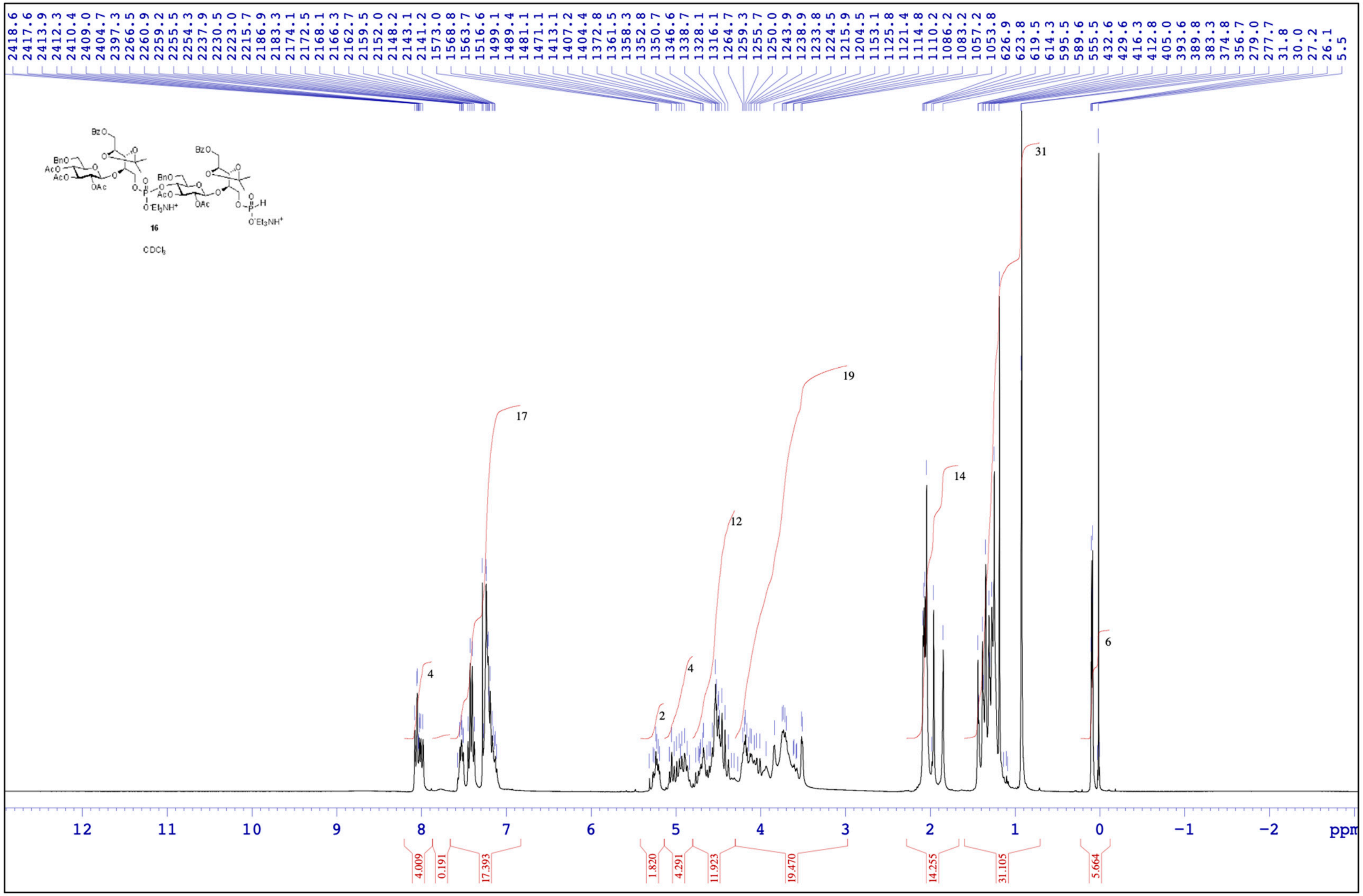

^{1}H NMR spectrum of compound **16** (300 MHz, $CDCl_3$)

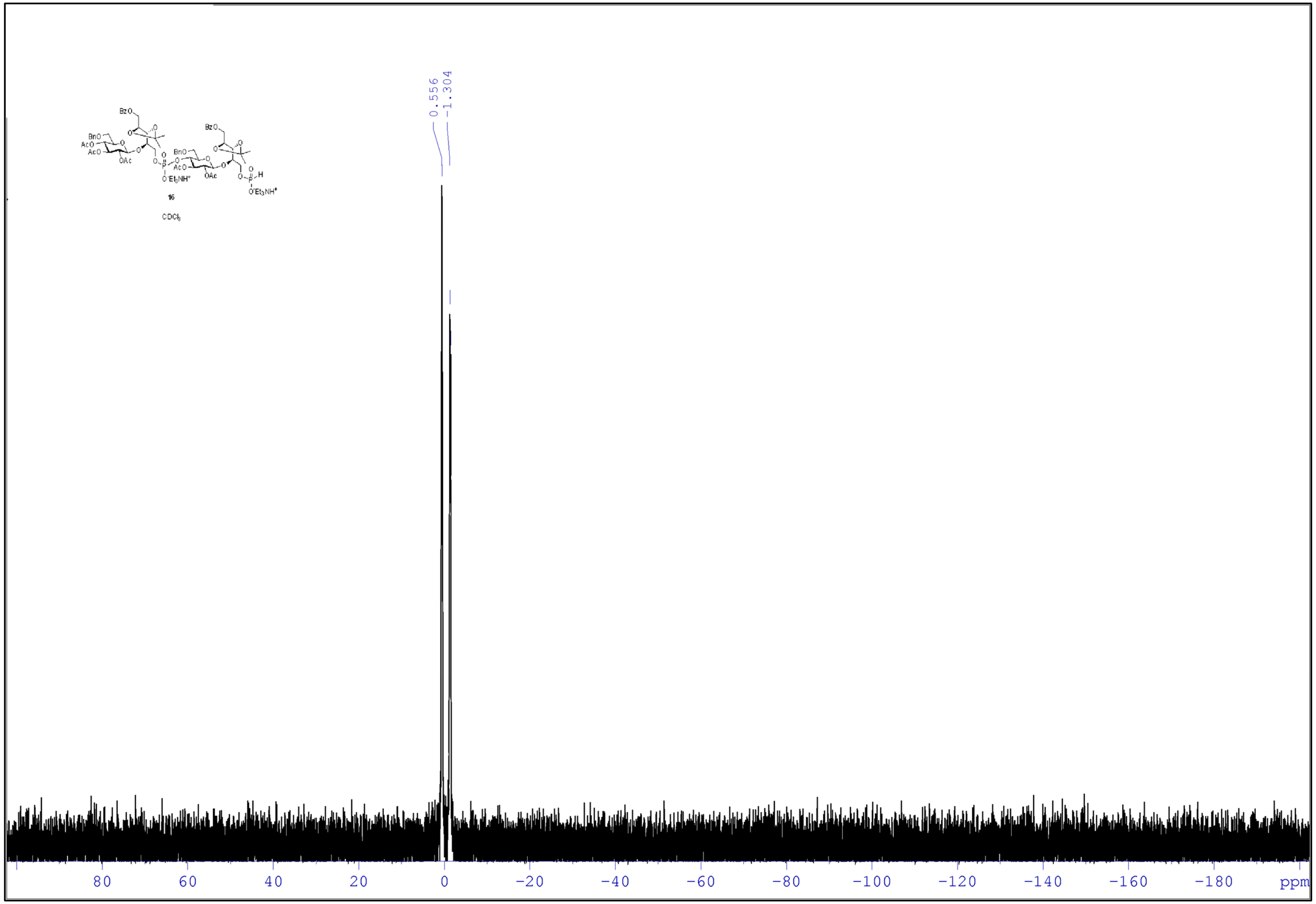

^{31}P NMR spectrum of compound **16** (122 MHz, $CDCl_3$)

9. ^{1}H and ^{31}P NMR Spectra of compound 17

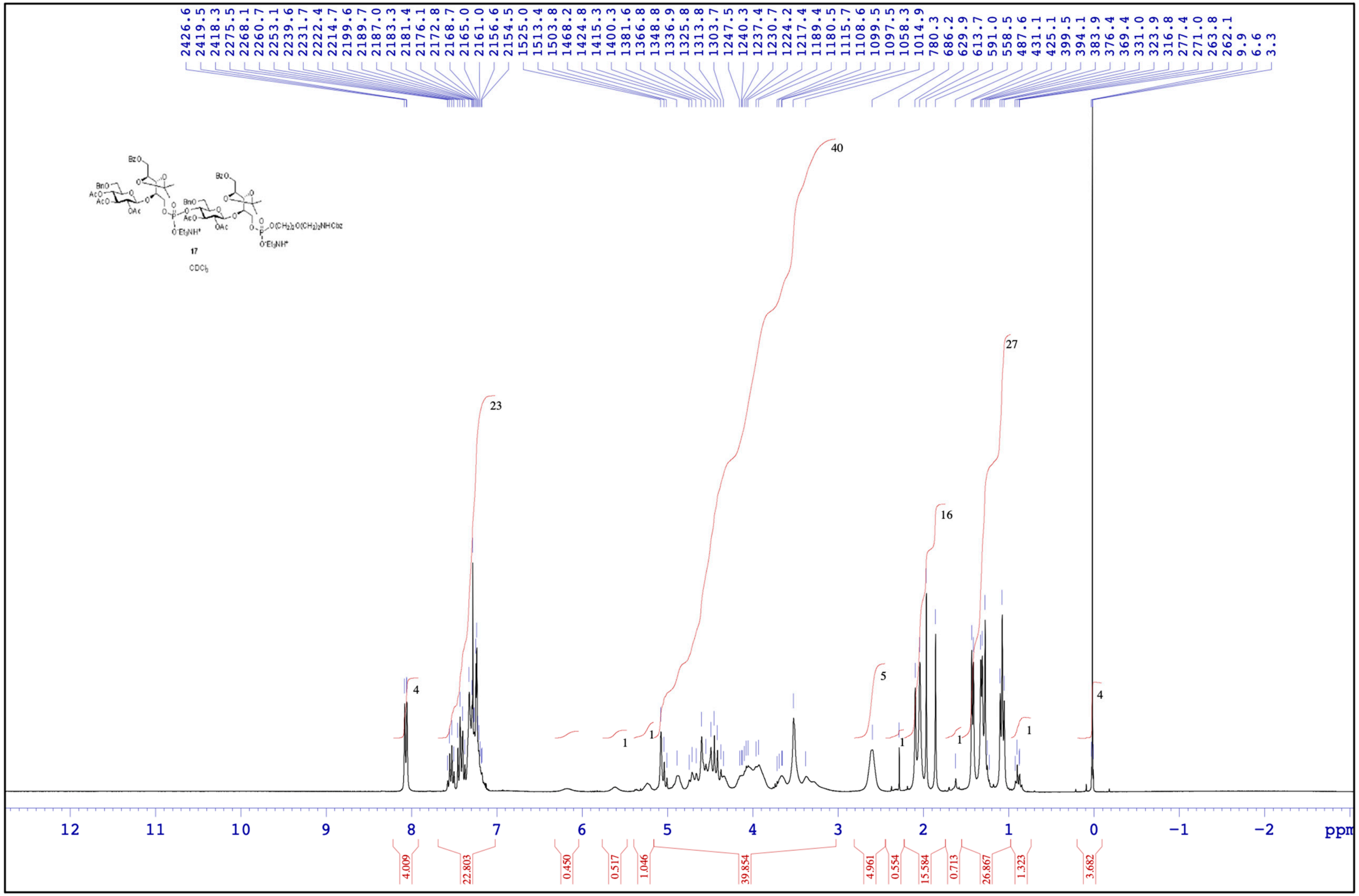

^{1}H NMR spectrum of compound **17** (300 MHz, $CDCl_3$)

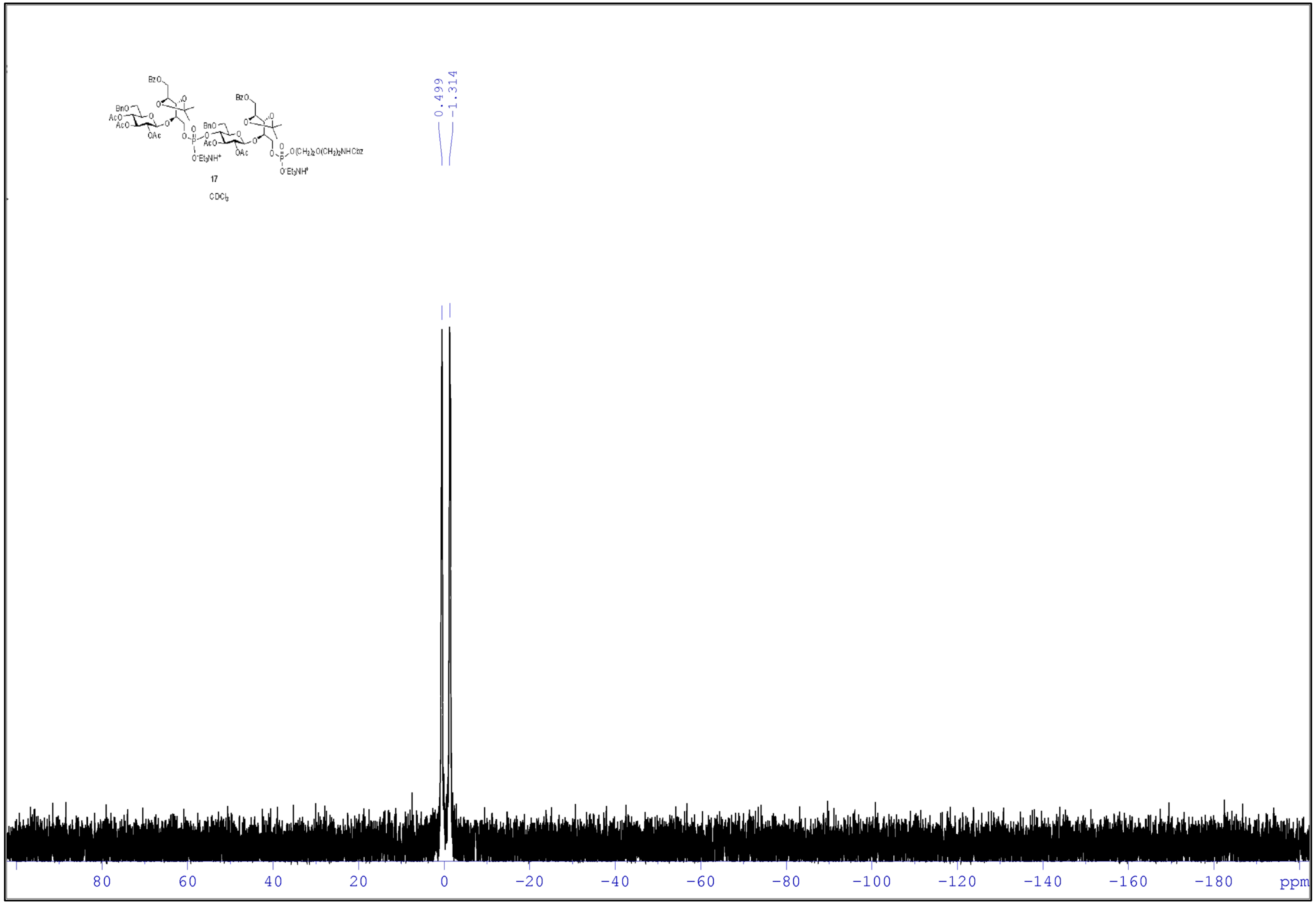

^{31}P NMR spectrum of compound **17** (122 MHz, $CDCl_3$)

10.^{1}H, ^{13}C and ^{31}P NMR Spectra of compound 1

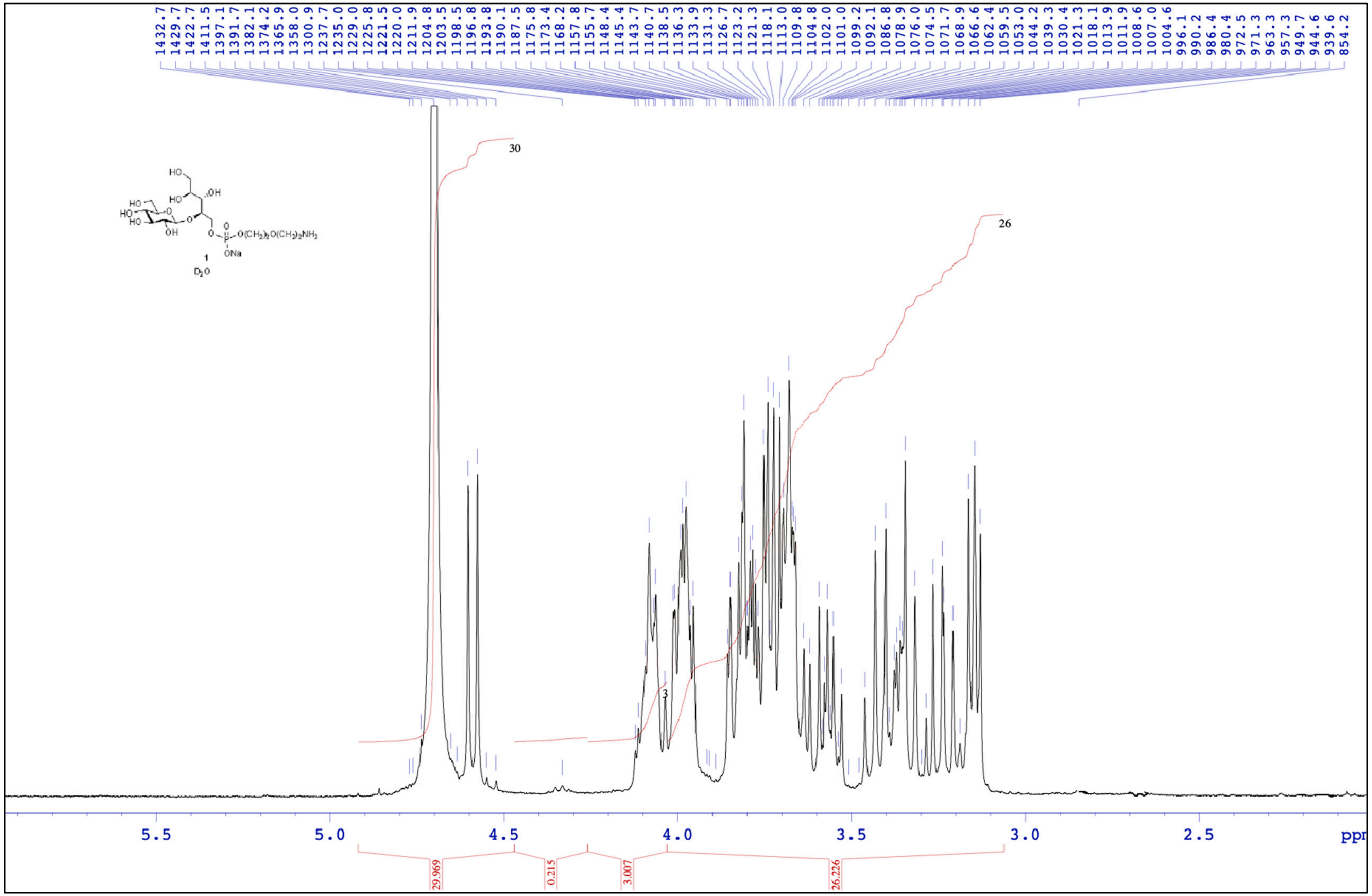

^{1}H NMR spectrum of compound **1** (300 MHz, D_2O)

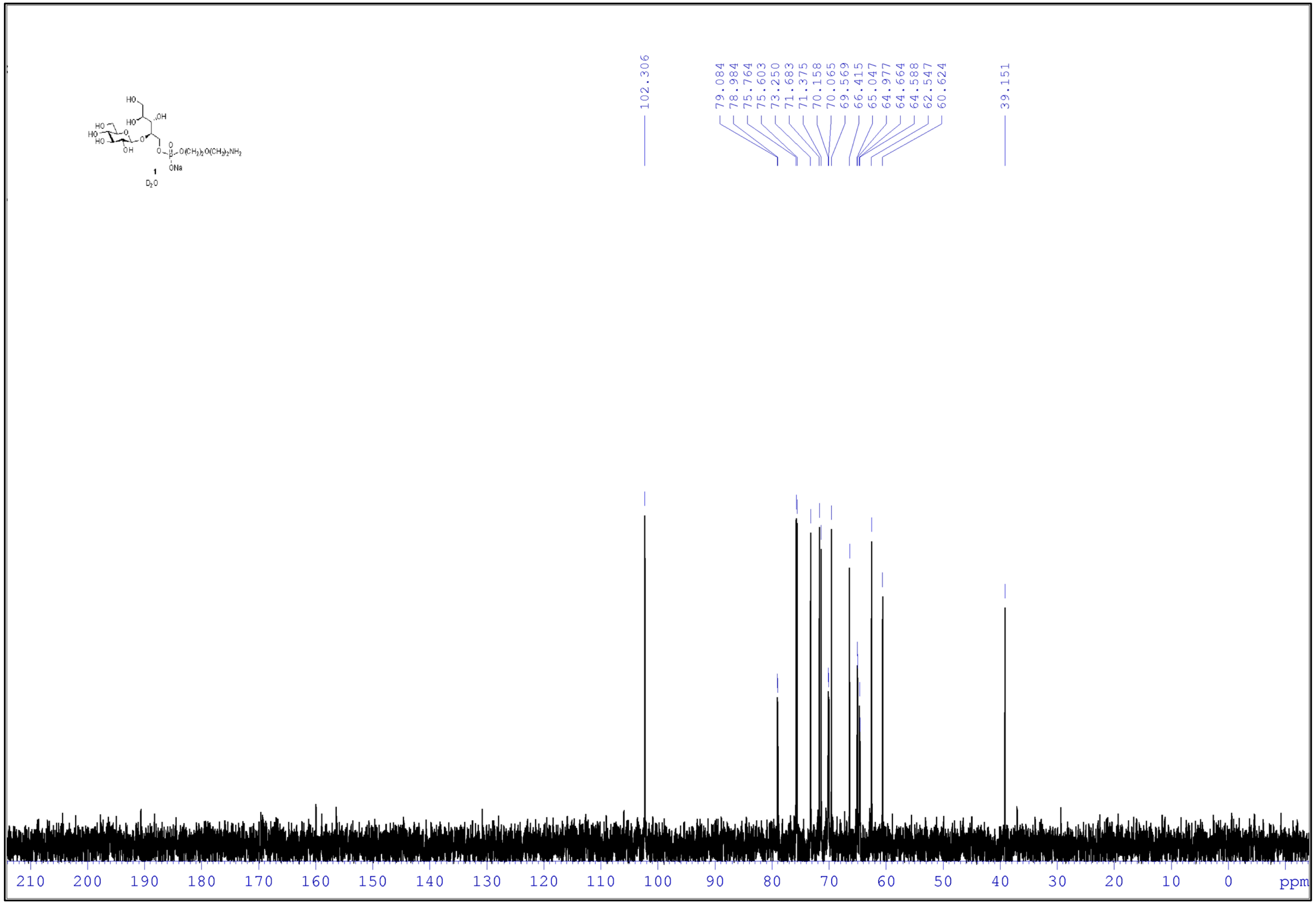

^{31}C NMR spectrum of compound **1** (75 MHz, D_2O)

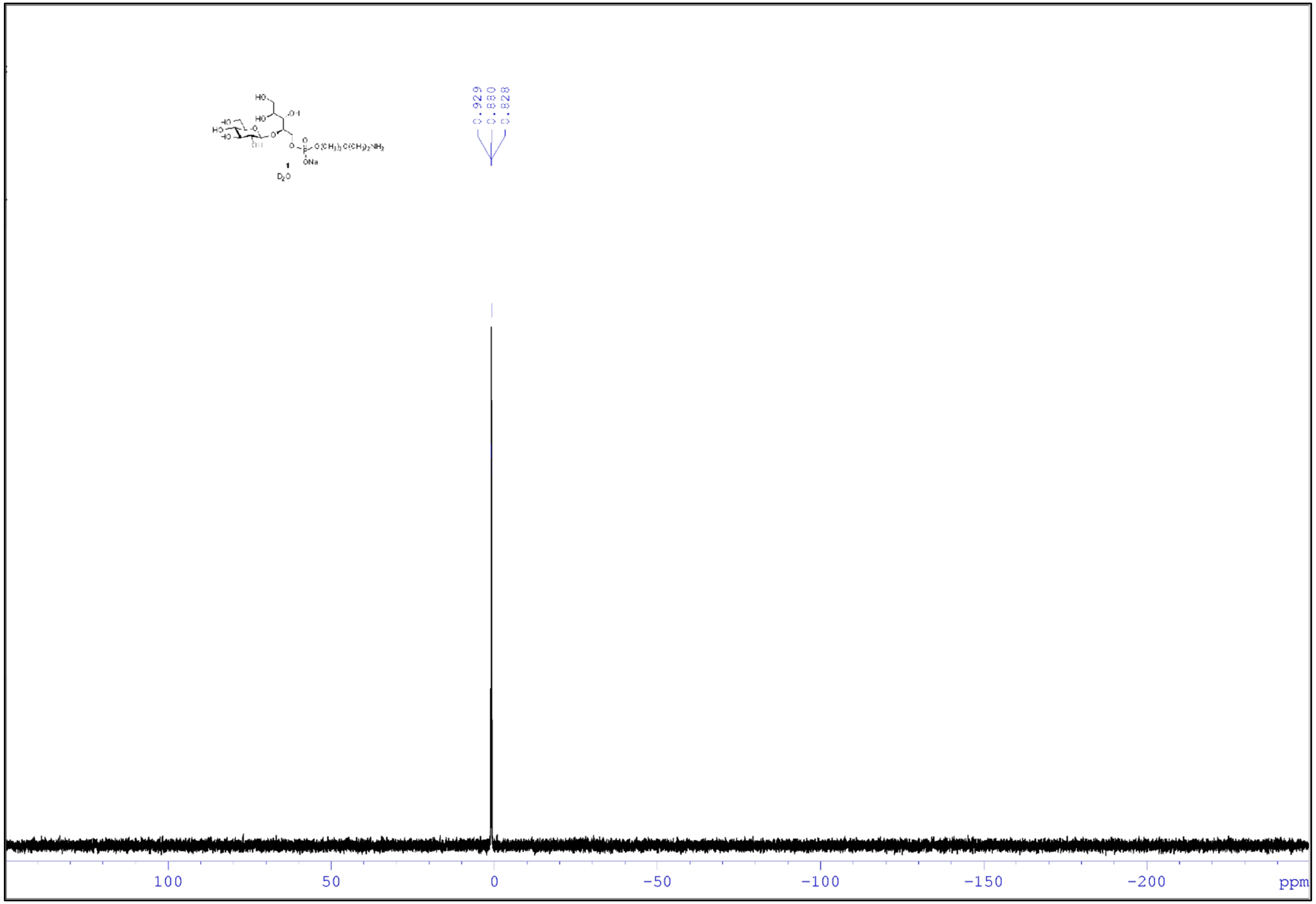

^{31}P NMR spectrum of compound **1** (122 MHz, D_2O)

11. ^{1}H, ^{13}C and ^{31}P NMR Spectra of compound 2

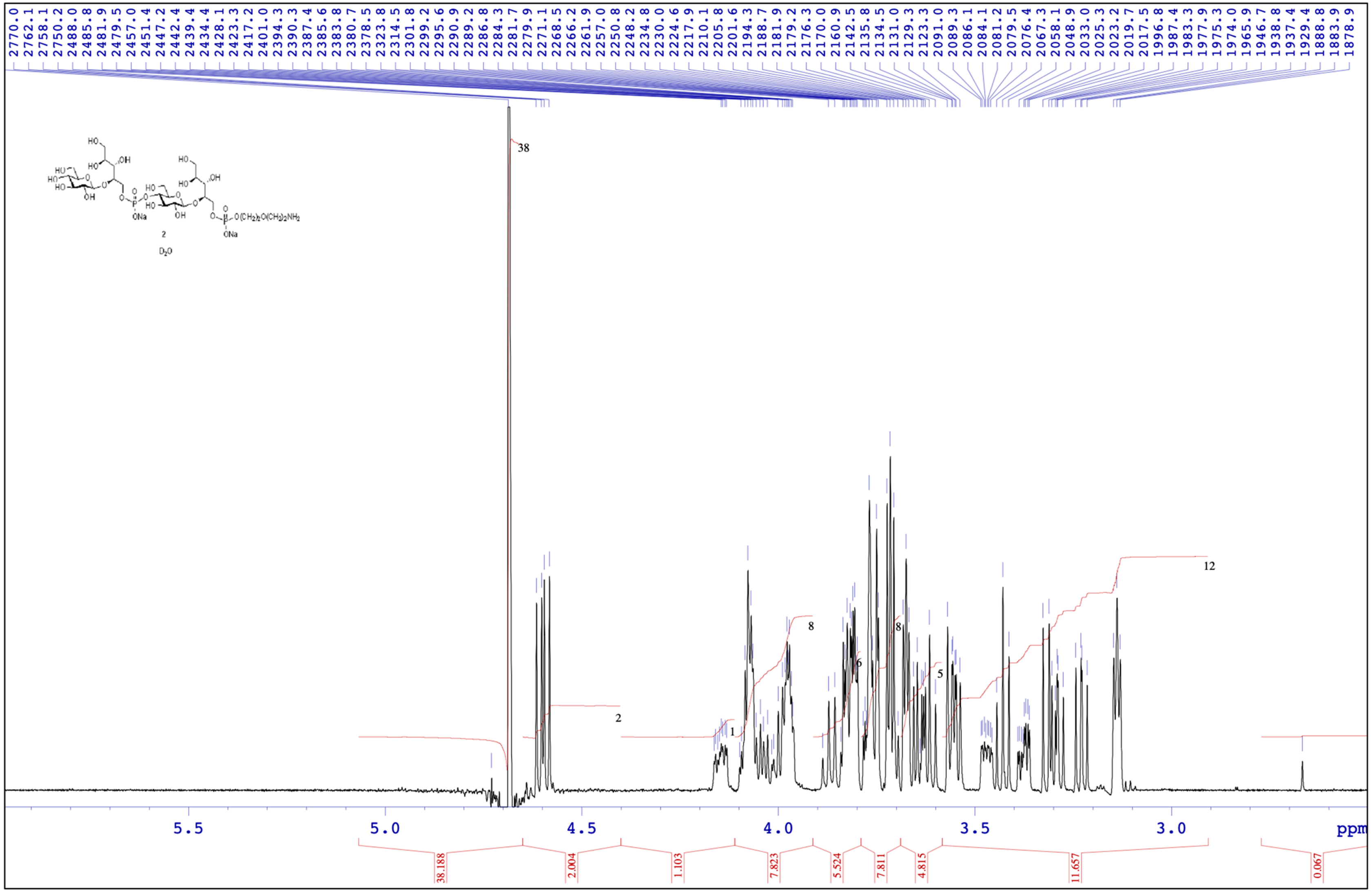

^{1}H NMR spectrum of compound **2** (600 MHz, D_2O)

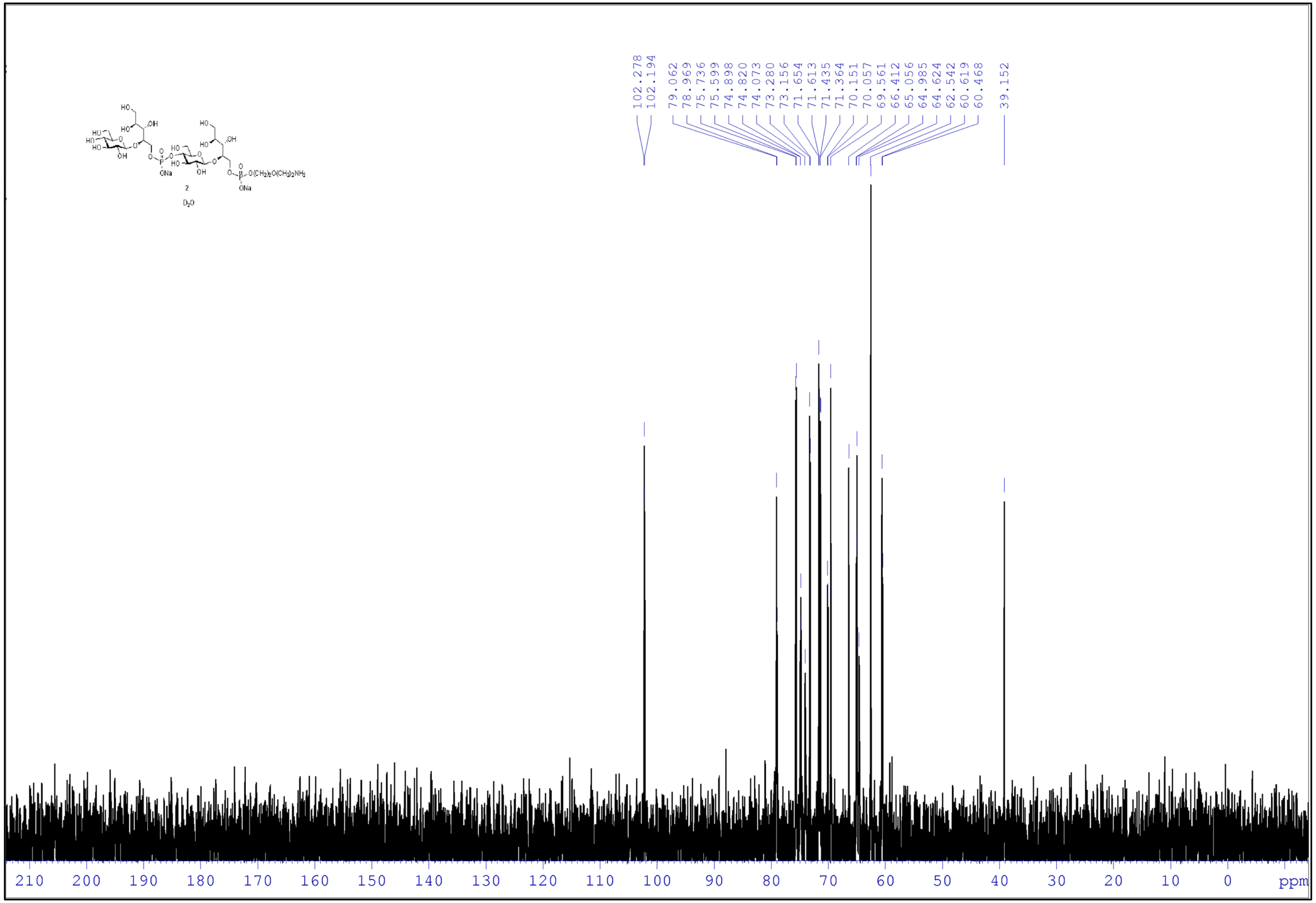

[31]C NMR spectrum of compound **2** (75 MHz, D_2O)

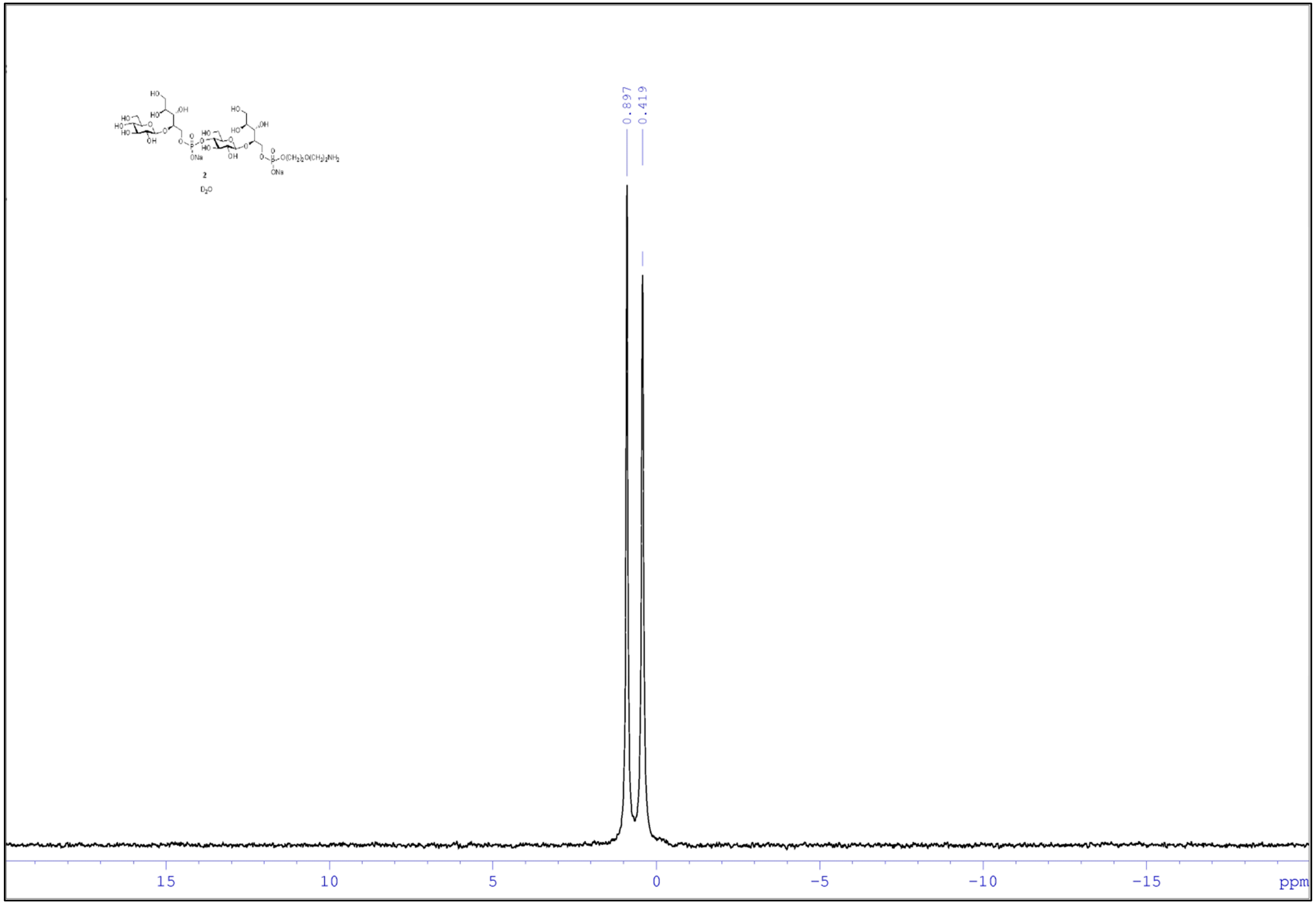

^{31}P NMR spectrum of compound **2** (243 MHz, D_2O)

12. ^{1}H, ^{13}C and ^{31}P NMR Spectra of compound 3

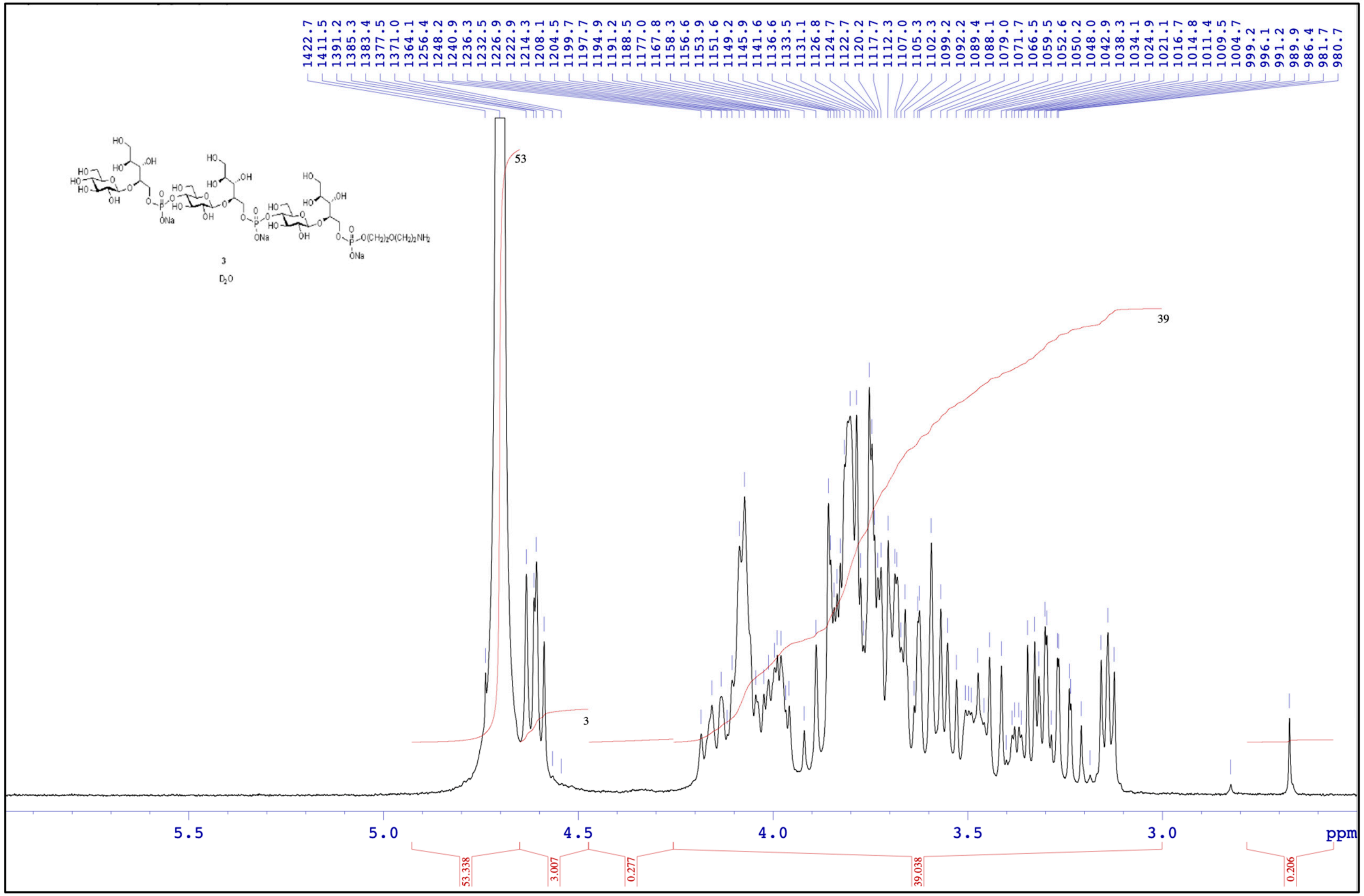

^{1}H NMR spectrum of compound **3** (300 MHz, D_2O)

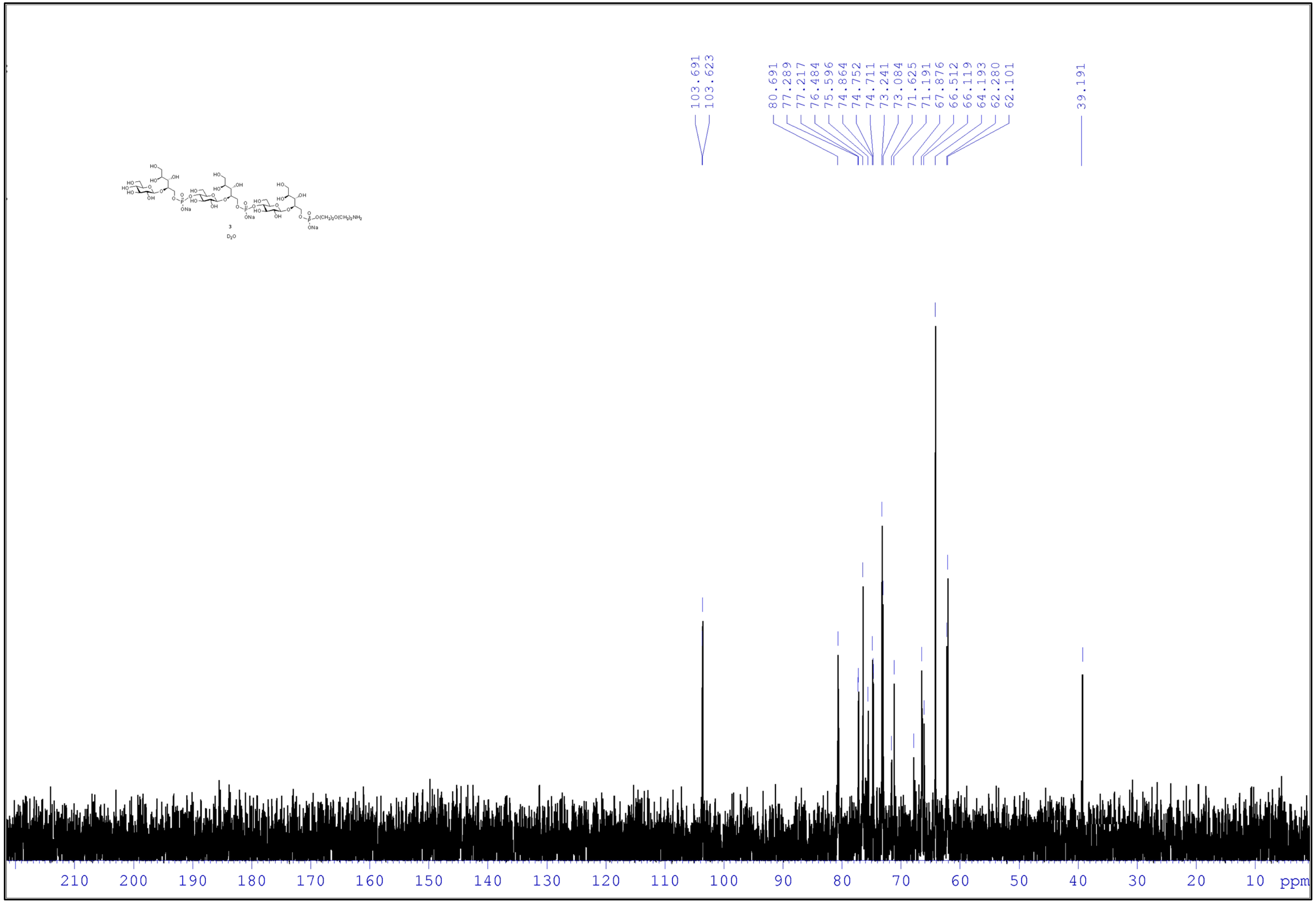

[31]C NMR spectrum of compound **3** (150 MHz, D_2O)

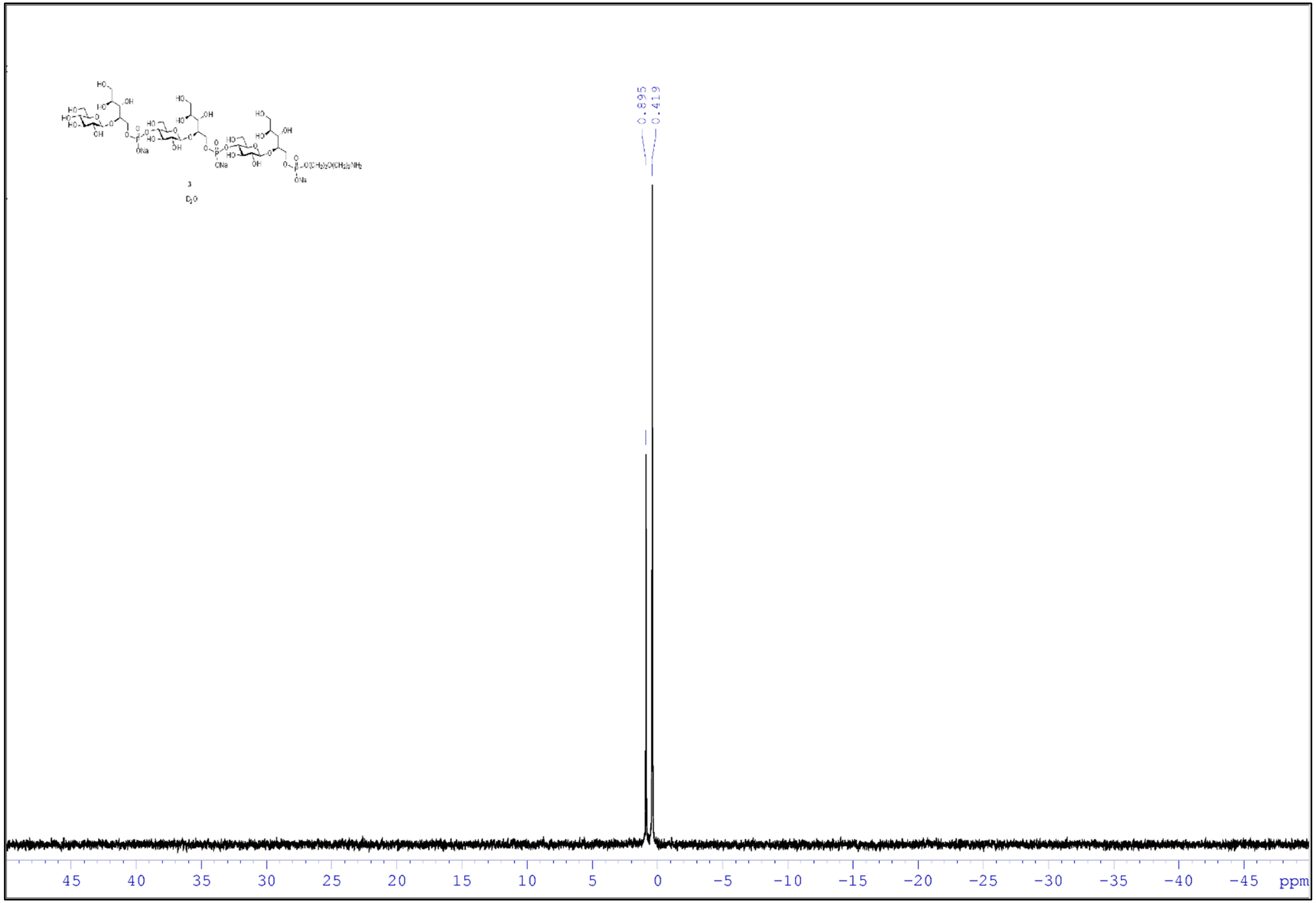

^{31}P NMR spectrum of compound **3** (122 MHz, D_2O)

13.^{1}H NMR Spectrum of compound 4

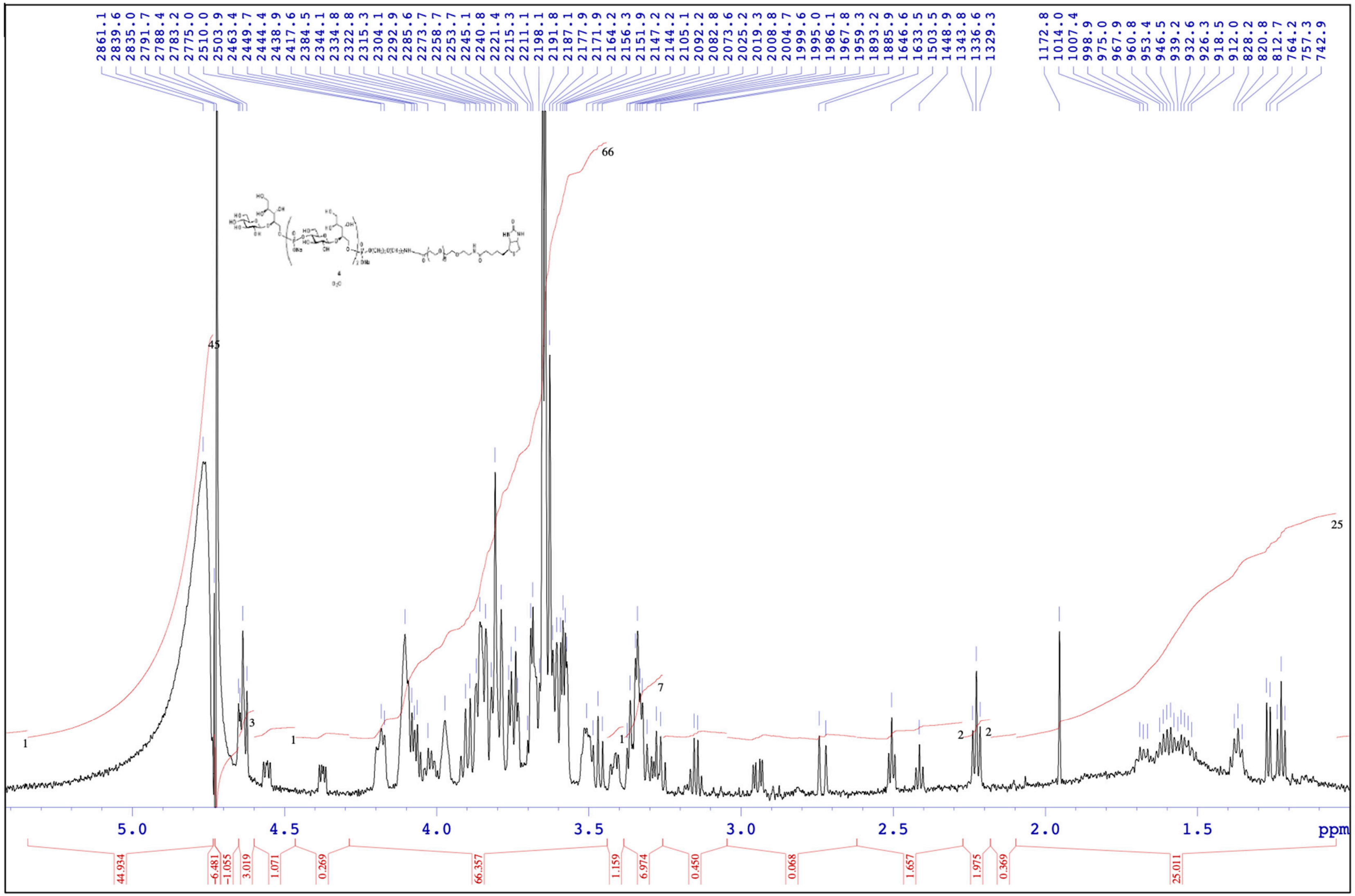

^{1}H NMR spectrum of compound **4** (600 MHz, D_2O)
